# Comparative evaluation of extraction methods for fragrant semen Trichosanthis oil: Cold pressing, conventional solvent, subcritical *n*-butane and supercritical CO_2_

**DOI:** 10.1016/j.fochx.2025.102670

**Published:** 2025-06-16

**Authors:** Ling-Biao Gu, Qiao-Ying Song, Lin Wang, Xue-Xia Liu, Wen-Jie Liao, Rong Gu, Hua-Min Liu, Ya-Ting Zhang, Kun-Peng Zhang, Tian-Xuan Hao

**Affiliations:** a Henan Key Laboratory of Subcritical High-Efficiency Extraction, School of Biological and Food Engineering, Anyang Institute of Technology, 455000 Anyang, China; b College of Food Science and Engineering, Henan University of Technology, 450001 Zhengzhou, China; c Library, Zhengzhou University, 450001 Zhengzhou, China; d College of Safety Science and Engineering, Henan Polytechnic University, 454000 Jiaozuo, China; e Henan Subcritical Biotechnology Co. Ltd., 455000 Anyang, China

**Keywords:** Fragrant semen Trichosanthis oil, Extraction methods, Physicochemical properties, Chemical composition, Volatile organic compounds, Gas chromatography-ion mobility spectrometry

## Abstract

The physicochemical properties, chemical compositions, antioxidant activities, and volatile profiles of fragrant Semen Trichosanthis oil (FSTO) extracted by cold pressing (CP), solvent extraction (SE), subcritical *n*-butane extraction (SBE), and supercritical CO_2_ extraction (SPE) were systematically investigated. Unsaturated fatty acids predominated in the oils (89.57–92.73 %), primarily trichosanic acid, linoleic acid, and oleic acid, with SBE oil containing the highest proportion (92.73 %). CP oil showed the highest phytosterol content (434.72 mg/100 g) and total flavonoid content, alongside the strongest antioxidant activity. In contrast, SBE oil had the greatest total phenolic content (62.08 μg GAE/g) and oxidative stability (1.24 h). All oils exhibited Newtonian flow behavior, with SBE and SPE oils exhibiting the lowest activation energy. A total of 108 volatile organic compounds were identified by gas chromatography-ion mobility spectrometry, with SBE and SPE oils displaying more complex and abundant profiles, while CP oil better retained natural aroma compounds. These results demonstrate that extraction method significantly influences the composition, functionality, and volatile profile of FSTOs. SBE achieved a favorable balance between oil yield, quality, and sustainability.

## Highlights

• Four extraction methods for fragrant Semen Trichosanthis oils (FSTOs) were compared.
• FSTOs are rich in UFAs, particularly trichosanic acid and linoleic acid.
• Cold pressing oil exhibited the highest phytosterol content and antioxidant activity.
• Subcritical *n*-butane extracted oil had the highest phenolics and oxidative stability.
• Gas chromatography-ion mobility spectrometry identified 108 volatile compounds.

## 1 Introduction

The increasing interest in non-traditional oilseed crops is driven by their potential to strengthen food security, provide health benefits, and diversify the global oilseed industry (Miao et al., 2022). Among these emerging crops, *Trichosanthes kirilowii* Maxim has attracted considerable attention due to its applications in traditional medicine, nutrition, and high-quality oil production (Hou et al., 2020). *Trichosanthes kirilowii* Maxim, commonly known as *Gua*-*lou* in China, is a perennial vine of the *Cucurbitaceae* family. Its fruits, seeds, and roots have been widely used in traditional Chinese medicine for nearly a thousand years (Zhang et al., 2023). Semen Trichosanthis (ST), the dried ripe seeds of *Trichosanthes kirilowii* Maxim, have been traditionally used to treat cough, inflammation, and constipation (Xu et al., 2022). ST possesses outstanding nutritional properties, making it highly valuable for edible oil production and functional food development. The edible seed kernels contain 38.06–54.23 % oil, primarily composed of unsaturated fatty acids (86.99–93.12 %), such as oleic acid, conjugated linoleic acid, and conjugated linolenic acid (Xu et al., 2022; Zhang et al., 2023). Both conjugated linoleic acid and conjugated linolenic acid provide various health benefits, including anti-cancer effects, tumor suppression, promotion of weight loss, immune enhancement, and regulation of blood lipids and metabolism (Du et al., 2024).

Traditionally, ST has been utilized as a snack—commonly known as *Guazi*—in the Chinese food industry. This snack is produced by roasting edible seeds. Roasted ST, referred to as *Diao-Guazi* in Chinese, exhibits a much more intense flavor than raw seeds and is employed both as a food product and in medicinal formulations. The volatile components of raw and roasted ST have been analyzed, revealing a significant decrease in sesquiterpenes and short-chain aldehydes as a result of roasting. Conversely, roasting resulted in higher levels of 3-methylbutanal, ethanol, 2-butanol, and alkyl pyrazines, contributing to the distinctive flavor of roasted ST (Wu et al., 2014).

An appropriate oil extraction method is essential for enhancing oil quality and preserving higher levels of bioactive compounds. Among traditional methods, cold pressing (CP) is environmentally friendly and avoids solvent pollution (Miao et al., 2022). It involves mechanically pressing seeds to extract oil, yielding pure oils that can be consumed without further refinement. This process effectively preserves the inherent properties of the raw material; as only minor components are affected. However, its oil recovery is low and bioavailability is limited, resulting in resource inefficiency (Sumara et al., 2023). Solvent extraction (SE), particularly using *n*-hexane, has proven to be an effective method, achieving oil extraction rates exceeding 90 %. It is widely employed in large-scale seed oil production, often serving as a supplement to mechanical pressing to increase yield. However, residual solvent in crude oil must be removed at high temperatures, which leads to the loss of heat-sensitive bioactive nutrients and diminishes the oil's nutritional value (Gu et al., 2019). Moreover, the process incurs higher operational costs than CP, primarily due to increased solvent consumption and energy-intensive processing. Consequently, there is an urgent need for an alternative, environmentally friendly solvent that can be easily removed under mild conditions, thereby preserving bioactive components and ensuring the stability and quality of the extracted oil.

In recent years, supercritical fluid extraction (SPE) and subcritical fluid extraction (SBE) have garnered significant attention as innovative extraction methods. SPE, primarily utilizing supercritical carbon dioxide, is an environmentally friendly and highly selective technique known for its exceptional yield and high-quality oils (Liu et al., 2023). The dissolving power of the SPE solvent can be adjusted by varying pressure and temperature. Supercritical fluids exhibit higher diffusion coefficient and lower viscosity and surface tension than liquid solvents, thereby enhancing mass transfer properties. Consequently, SPE is widely employed for oil extraction and the rapid separation of target compounds from natural products. However, the widespread application of SPE is limited by high equipment and operating costs due to the extremely high pressure required, challenges in continuous production, and its inability to meet large-scale industrial demands (Gu et al., 2016).

SBE, an eco-friendly technology operating at lower pressure and energy, offers significant advantages over traditional solvent extraction methods, including enhanced safety, efficiency, and suitability for large-scale industrial applications (Gu et al., 2016). Compressed *n*-butane is one of the most commonly used solvents employed in SBE for lipid extraction, remains in a liquid state between its boiling point (−0.50 °C) and critical temperature (152.01 °C) (Hou et al., 2024). Under subcritical conditions, solvents exhibit reduced viscosity and density, along with enhanced diffusivity and solubility, enabling the efficient extraction and separation of heat-sensitive compounds while minimizing oxidation (Wang et al., 2021). *n*-Butane can be easily removed through system depressurization at low temperatures and subsequently recycled after extraction. Moreover, *n*-butane is cost-effective, colorless, and has low biological toxicity (Gu et al., 2019). Consequently, it is legally approved for use as a food processing aid in China and Europe.

Previously, various methods—CP, ultrasonic-assisted solvent extraction (UASE), SPE, and SBE—have been used to extract oils from ST (Xu et al., 2022). It has been reported that different extraction methods influence the quality, lipid composition, and antioxidant capacity of ST oil. Moreover, oil extracted from de-shelled seeds contains higher levels of tocopherol and phytosterol, along with greater free radical scavenging activity. These results indicate that extraction methods are crucial for the final oil product, and that ST oil holds significant application potential. To date, no studies have reported the impact of various extraction methods on the physicochemical and functional properties of fragrant ST oil (FSTO), and the literature offers only limited information on its volatile compounds.

Therefore, the primary objective of this study is to comprehensively compare the effects of four extraction methods (CP, SE, SBE, and SPE) on the physicochemical properties, fatty acid composition, bioactive compounds, oxidative stability, rheological behavior, volatile organic compounds (VOCs), and in vitro antioxidant activity of FSTO. This study provides valuable insights into the future application of ST in flavored edible oils and the development of functional, oil-based products.

## 2 Materials and methods

### 2.1 Materials and chemicals

ST was collected from a regional farm in Anyang, China. The raw seeds (100 g) were roasted in a 500 mL glass laboratory roaster equipped with an oil bath and a magnetic stirrer. The optimal roasting condition was determined to be 150 °C for 30 min based on aroma impressions assessed by 10 panelists. The roasted seeds were husked, ground into powder, and sieved through a 40-mesh mechanical sieve.

Fatty acid methyl ester, tocopherol and phytosterol standards, along with *n*-hexane, isopropanol, rutin, gallic acid, DPPH, and ABTS, were obtained from Sigma-Aldrich (St. Louis, MO, USA). *n*-Butane was purchased from Puyang Longyu Chemical Co., Ltd. (Henan, China). Additional reagents and solvents were obtained from Baoman Biotechnology Co., Ltd. (Shanghai, China).

### 2.2 Methods

#### 2.2.1 Oil extraction

CP was performed using a hydraulic press (XL-600, Bafang Co., Ltd., Zhengzhou, China) at 60 MPa and a temperature of 40 °C for a duration of 1.5 h.

SE was carried out using an SER 148/6 solvent extractor (Velp Scientifica, Usmate, Italy). Ten grams of dried seed powder were accurately weighed and wrapped in a filter paper thimble. The thimble was then placed into the extractor, and 60 mL of *n*-hexane was added as the solvent. The immersion, washing, and recovery steps were carried out at 70 °C for 1, 3, and 1 h, respectively.

SBE was performed using an extraction device (CBE-5 L, Henan Subcritical Biological Technology Co., Ltd., Anyang, China). For each extraction, 100 g of powdered seed mixture was placed into the extractor, which was then vacuumed to eliminate atmospheric oxygen that could cause oil oxidation during extraction. Subsequently, 1000 mL of subcritical *n*-butane was introduced into the extractor. The extraction was carried out at 40 °C for 30 min over 3 cycles. At the end of extraction, the subcritical *n*-butane containing the lipid was transferred to a separator, and after the solvent was removed by reduction vaporization, the extracted oil was collected.

SPE was performed using a SPE device (HA120–50-01, Yi Chuang Experimental Instrument Co. Ltd., Nantong, China). For each extraction, 100 g of powdered seed mixture was placed into a 1 L extractor and preheated at 30 °C. The extraction pressure (25 MPa), temperature (40 °C), and CO_2_ flow rate (10 L/h) were controlled using regulating valves. Supercritical CO_2_ was injected into the bottom of the extractor and exited through the top outlet valve along with the extracted oil, directed to a separator. In the separator, the CO_2_ was depressurized to gas form, and the oil was collected through the bottom outlet valves.

The optimal conditions for CP, SE, SBE, and SPE were established based on preliminary laboratory experiments. The extracted oils were collected, and residual solvents were removed using a rotary vacuum evaporator. The oils were then weighed and stored at 4 °C, with yields calculated as the mass of extracted oil to initial sample weight.

#### 2.2.2 Physicochemical properties

The AOAC Official Methods Cd 3d–63, 993.20, 965.33, and 920.160 were applied to determine the acid value (AV), iodine value (IV), peroxide value (POV), and saponification value (SV), respectively. An Abbe refractometer (2 W, Shanghai Csoif Co., Ltd., Shanghai, China), a 10.0 mL specific gravity bottle, and a spectrophotometer (CS–821 N, Hangzhou Color Spectrum Technology Co., Ltd., China) were used to measure the refractive index, specific gravity, and color values (L*, a*, and b*) at room temperature, respectively.

#### 2.2.3 Determination of fatty acid

The fatty acid profiles of the obtained oils were analyzed using a gas chromatography system coupled to a flame ionization detector (7890B, Agilent Co., Santa Clara, CA, USA) and an HP-88 capillary column (100 m × 0.25 mm × 0.20 μm, Agilent Co., Santa Clara, CA, USA), according to the AOAC Official Method Ce 1–62. Before GC analysis, the oils were converted into fatty acid methyl esters following the procedure reported by Gu et al. (2016). The fatty acid composition was expressed as relative content.

#### 2.2.4 Determination of bioactive compounds

The tocopherol content was determined using an e2695 HPLC system (Waters, Milford, MA, USA) equipped with a 2475 fluorescence detector. A mixture of *n*-hexane and isopropanol (99,1, *v*/v) was used as the mobile phase at a flow rate of 1 mL/min. The column temperature was maintained at 35 °C, and the excitation and emission wavelengths were set at 294 nm and 328 nm, respectively. Individual compounds were identified and quantified using external standard methods.

The phytosterol composition was analyzed using a gas chromatography system (7890B, Agilent Co., Santa Clara, CA, USA) equipped with a flame ionization detector and an HP-5 column (30 m × 0.32 mm × 0.25 μm, Agilent Co., Santa Clara, CA, USA). The analysis was conducted according to the ISO 12228-1:2014 method. The GC conditions were adopted from a previous study (Qin et al., 2024). Individual compounds were identified by matching their retention times with those of standard phytosterols, and quantitative analysis was subsequently performed using the internal standard method with 5α-cholestan-3β-ol as the internal standard.

The total phenolic content (TPC) and total flavonoid content (TFC) were measured using the Total Phenol Content Assay Kit and Total Flavonoids Content Assay Kit (Megazyme, Bray, Ireland), following the manufacturer's instructions. Results are expressed as gallic acid equivalents (μg GAE/g) and rutin equivalents (μg RE/g).

#### 2.2.5 Oxidative stability

The oxidative stability of 3.0 ± 0.1 g obtained oils was evaluated using a Rancimat apparatus (743, Metrohm, Switzerland) at 100 °C with an O_2_ flow rate of 20 mL/h. The induction time (IT) was recorded and reported in hours.

#### 2.2.6 Rheological measurements

Rheological measurements were conducted following our previously reported method (Gu et al., 2019), utilizing an MCR 102 rheometer (Anton Paar, Graz, Austria) equipped with a concentric cylinder (CC24) of 27 mm inner diameter. Flow curves were generated across a shear rate range of 1–1000 s^−1^ at 25 °C, and a temperature sweep test was performed within a range of 5–100 °C. Rheological data were collected using Rheoplus software version 3.21 (Anton Paar, Graz, Austria). Subsequently, the obtained data were fitted to the Arrhenius equation (Eq. (1)):

(1) $$\mu ={\mathrm{Ae}}^{\left(-E_{a}/\mathrm{RT}\right)}$$

where *μ* is the viscosity (Pa·s), A is the Arrhenius constant, *E*_a_ is the activation energy (kJ/mol), R is the universal gas constant (8.314 J/mol/K), and *T* is the temperature (K).

#### 2.2.7 In vitro antioxidant activity

The DPPH radical scavenging activity was evaluated following a previously described method (Gu et al., 2019) with slight modifications. Briefly, 1 mL of the sample solution was mixed with 4 mL of freshly prepared DPPH solution (0.1 mM in ethanol). The mixture was thoroughly shaken and incubated in the dark at room temperature for 30 min. Subsequently, the absorbance at 517 nm was measured against a blank containing all reagents except the sample.

For ABTS radical scavenging, the ABTS radical cation was produced by reacting a 7 mM ABTS solution with a 2.45 mM potassium persulfate solution, followed by incubation in darkness at room temperature for 16 h. The resulting ABTS radical solution was then diluted with ethanol to achieve an absorbance of 0.70 ± 0.02 at 734 nm. Then, 1 mL of the sample solution was combined with 4 mL of the diluted ABTS radical solution and incubated at room temperature for 15 min. The absorbance at 734 nm was then measured against a blank containing all reagents except the sample.

The scavenging rates of DPPH and ABTS radicals were calculated using the following equation (Eq. (2)):

(2) $$\text{Scavenging rate}\;\left(\%\right)=\left(A_{\mathrm{blank}}-A_{\text{sample}}/A_{\mathrm{blank}}\right)\times 100$$

The IC_50_ value, defined as the oil concentration (mg/mL) required to scavenge 50 % of DPPH or ABTS free radicals, was calculated based on fitted scavenging curves generated using GraphPad Prism 8.2.1 (GraphPad Software, San Diego, USA).

#### 2.2.8 VOCs analysis

The detection of VOCs was performed using a Gas chromatography-ion mobility spectrometry (GC-IMS) system (Flavourspec®, G.A.S, Dortmund, Germany) equipped with a WAX capillary column (15 m × 53 mm × 1 μm, RESTEK Company), as described by Xu, Wang, et al. (2023). Approximately 2 g of sample was incubated at 80 °C for 20 min in a 20 mL headspace vial at 500 rpm. A 500 μL headspace sample was injected using an auto-sampler with a heated syringe at 85 °C. Column and IMS temperatures were set at 60 °C and 45 °C, respectively. High-purity nitrogen served as the carrier gas. The carrier gas flow program started at 2 mL/min (2 min), increased to 10 mL/min (10 min), then to 100 mL/min (20 min), and held for 20 min. Drift gas flow rate was kept at 150 mL/min. Spectra were averaged over 12 scans. Compounds were identified by their retention index (RI) and drift time (DT) using the GC-IMS library. Signal intensities from LAV software were used for relative quantification.

### 2.3 Statistical analyses

The collected data were statistically analyzed utilizing SPSS Statistics software version 19.0 (SPSS Inc., Chicago, IL, USA). Significant differences among the oils were determined using analysis of variance (ANOVA) followed by Duncan's multiple range test at a 95 % confidence level (α = 0.05). All results are presented as the arithmetic means of three independent measurements ± standard deviation (SD).

## 3 Results and discussion

### 3.1 Extraction yields and physicochemical characterization of oils extracted using various methods

The yields and physicochemical characteristics of FSTOs obtained through various extraction methods are presented in Table 1. SE yielded the highest FSTO extraction at 36.26 %, consistent with previous reports (Xu et al., 2022; Zhang et al., 2023). CP, SBE, and SPE achieved 79.16 %, 96.94 %, and 92.15 % of the SE yield, respectively. This suggests that SE and SBE are more effective for extracting plant oils. However, CP was limited by lower oil yields and bioavailability, leading to resource waste. Additionally, SE requires a longer processing time (300 min) compared to SBE (90 min). Furthermore, the high operational costs of SPE, due to the high pressure (∼30 MPa), limit its application in the food industry. Conversely, subcritical *n*-butane is a suitable solvent for low-pressure oil extraction (∼0.35 MPa), making it a more viable option for industrial applications (Gu et al., 2019). Beyond extraction yield, these methods vary in energy consumption and environmental impact. CP is energy-efficient and environmentally friendly, as it requires neither heat nor solvents (Miao et al., 2022; Sumara et al., 2023). SE yields high output but entails prolonged processing times and the use of organic solvents, which may pose environmental risks if not properly managed (Gu et al., 2019). SBE operates under mild conditions—low pressure and temperature—thereby reducing energy consumption and solvent residues. The food-grade *n*-butane used in SBE can be entirely removed through depressurization, enhancing environmental safety (Gu et al., 2016; Hou et al., 2024). SPE requires high pressure and substantial energy input, potentially increasing costs and environmental burden (Liu et al., 2023). These differences suggest that SBE offers a more balanced approach regarding yield, environmental impact, and industrial feasibility.

**Table 1:** Extraction yields, physicochemical properties and fatty acid composition of FSTOs extracted with different methods.

Paramaters	Samples			
	CP	SE	SBE	SPE
Extraction yield (%)	28.73 ± 0.41^d^	36.29 ± 0.36^a^	35.18 ± 0.45^b^	33.44 ± 0.38^c^
L* value	24.69 ± 0.03^d^	48.72 ± 0.03^b^	39.05 ± 0.03^c^	82.09 ± 0.01^a^
a* value	21.93 ± 0.01^a^	11.84 ± 0.02^c^	13.94 ± 0.05^b^	0.34 ± 0.02^d^
b* value	39.47 ± 0.01^d^	70.17 ± 0.02^b^	60.17 ± 0.02^c^	83.08 ± 1.16^a^
Specific gravity ($d_{20}^{20}$, g/mL)	0.916 ± 0.00^c^	0.915 ± 0.00^c^	0.921 ± 0.00^b^	0.924 ± 0.00^a^
Refractive index (*n*^20^)	1.62 ± 0.00^a^	1.61 ± 0.00^a^	1.61 ± 0.00^a^	1.60 ± 0.00^a^
Acid value (mg KOH/g)	0.57 ± 0.01^a^	0.47 ± 0.03^b^	0.50 ± 0.02^b^	0.49 ± 0.03^b^
Peroxide value (g/100 g)	0.24 ± 0.01^b^	0.27 ± 0.01^a^	0.21 ± 0.01^c^	0.09 ± 0.02^d^

Fatty acid profiles				
Palmitic acid (C16:0)	3.30 ± 0.02^b^	3.60 ± 0.01^a^	3.17 ± 0.00^c^	3.15 ± 0.00^c^
Stearic acid (C18:0)	2.41 ± 0.02^b^	2.53 ± 0.00^a^	2.37 ± 0.00^c^	2.41 ± 0.00^b^
Oleic acid (C18:1)	20.07 ± 0.09^b^	22.82 ± 0.05^a^	20.03 ± 0.02^b^	19.74 ± 0.01^c^
Linoleic acid (C18:2)	35.08 ± 0.10^b^	34.42 ± 0.05^c^	35.42 ± 0.02^a^	35.29 ± 0.01^a^
Linolenic acid (C18:3)	0.43 ± 0.00^c^	0.52 ± 0.00^a^	0.45 ± 0.00^b^	0.42 ± 0.00^d^
Trichosanic acid (9c, 11 t, 13c-C18:3)	37.01 ± 0.22^a^	34.42 ± 0.08^b^	37.28 ± 0.2^a^	34.54 ± 0.01^c^
α-Eleostearic acid (9c, 11 t, 13 t-C18:3)	1.16 ± 0.05^b^	1.15 ± 0.06^b^	0.88 ± 0.03^c^	3.2 ± 0.01^a^
Catalpic acid (9 t, 11 t, 13c -C18:3)	0.54 ± 0.07^b^	0.53 ± 0.09^b^	0.40 ± 0.13^b^	1.24 ± 0.00^a^
SFA	5.71 ± 0.04^b^	6.14 ± 0.01^a^	5.54 ± 0.00^c^	5.56 ± 0.00^c^
MUFA	20.07 ± 0.09^b^	22.82 ± 0.05^a^	20.03 ± 0.02^b^	19.74 ± 0.01^c^
PUFA	74.22 ± 0.13^c^	71.04 ± 0.06^d^	74.43 ± 0.02^b^	74.70 ± 0.01^a^
UFA	94.29 ± 0.04^b^	93.86 ± 0.01^c^	94.46 ± 0.01^a^	94.44 ± 0.01^a^

*Note*: ND: not detected; L* value: lightness; a* value: red-green variation (redness is positive, greenness is negative); b* value: yellow-blue change (yellow is positive, blue is negative); SFA: total saturated fatty acids; MUFA: total monounsaturated fatty acids; PUFA: total polyunsaturated fatty acid; UFA: total unsaturated fatty acids; TFA: total trans fatty acid. Different letters indicate significant differences at *P* < 0.05.

The color of edible oils is a critical sensory attribute that influences consumer acceptance (Qin et al., 2024). In this study, the L* values ranged from 24.69 (CP oil) to 82.09 (SPE oil), indicating that SPE yielded lighter oil of higher quality and purity. This method removes pigments, thereby enhancing oil brightness and visual appeal. The a* values ranged from 21.93 (CP oil) to 0.34 (SPE oil), indicating that CP oil exhibits a stronger red hue while SPE oil shows a shift toward green. This shift was attributed to the extraction conditions, particularly temperature and type of solvent used. Furthermore, the b* values increased from 39.47 (CP oil) to 83.08 (SPE oil), reflecting a more pronounced yellow hue in SPE oil. This enhancement might result from the increased solubility of certain yellow pigments during extraction.

The highest specific gravity was observed in SPE oil (0.924 g/mL), followed by SBE oil (0.921 g/mL). CP and SE oils exhibited similar specific gravities of 0.916 g/mL and 0.915 g/mL, respectively. These variations indicate that the extraction method may influence oil density, potentially due to differences in fatty acid composition and the presence of impurities. Refractive indices were consistent across all methods, ranging from 1.60 to 1.62, indicating that the extraction processes had no significant effect (*p* > 0.05). CP oil exhibited the highest acid value (0.57 mg KOH/g), indicating a higher level of free fatty acids compared to SE (0.47 mg KOH/g), SBE (0.50 mg KOH/g), and SPE (0.49 mg KOH/g). This suggests that the CP method may lead to increased lipid degradation or hydrolysis during extraction. Peroxide values varied significantly, with SPE oil showing the lowest value (0.09 g/100 g), followed by SBE (0.21 g/100 g), SE (0.27 g/100 g), and CP (0.24 g/100 g). SPE oil's lower peroxide value indicates reduced oxidation, likely due to high-pressure, low-temperature conditions that minimize the formation of oxidative products.

### 3.2 Fatty acid profiles of oils extracted using various methods

The fatty acid (FA) profiles of oils influence both their physical properties and nutritional value. Table 1 presents the fatty acid composition of FSTOs obtained using different methods. The results indicate that unsaturated fatty acids (UFAs) were predominant in all samples. Monounsaturated fatty acids (MUFAs) constituted 19.74–22.82 % of total FAs, while polyunsaturated fatty acids (PUFAs) accounted for 71.04–74.70 %. The fatty acid composition was comparable to a previous report (Yuan et al., 2009), though differences in content may be attributed to variations in cultivar, kernel maturity, geographical factors, and growth conditions.

The major UFAs—trichosanic acid (9c, 11 t, 13c-C18,3), linoleic acid (C18:2), and oleic acid (C18:1)—accounted for 89.57–92.73 % of total fatty acids. SBE oil exhibited the highest UFA content (92.73 %), followed by CP oil (92.16 %). Trichosanic acid (punicic acid), an isomer of conjugated linolenic acid (CLnA), is commonly referred to as “super CLnA” due to its enhanced bioactivity compared to conventional CLnA. It possesses various pharmacological properties, including antidiabetic, antiobesity, antiproliferative, and anticancer effects (Aruna et al., 2016). Linoleic acid (C18:2) and oleic acid (C18:1) together accounted for 52.20–62.51 % of total FAs. These fatty acids play a crucial role in human health, particularly in cholesterol reduction and prevention (Sumara et al., 2023).

α-Eleostearic acid (0.88 %–3.20 %) and catalpic acid (0.40 %–1.24 %) are additional conjugated trienoic fatty acid isomers of CLnA. α-Eleostearic acid exhibits stronger inhibitory effects on breast cancer cell proliferation than trichosanic acid (Kobori et al., 2008). Catalpic acid lowers fasting blood glucose and insulin levels in obese mice while enhancing glucose normalization following stimulation (Du et al., 2023). Additionally, the relative content of linolenic acid (C18:3), an essential PUFA, varied significantly, ranging from 0.42 % in SPE oil to 0.52 % in SE oil. Linolenic acid is a bioactive compound with substantial practical value, providing numerous health benefits, such as cardiovascular protection, diabetes management, antioxidant effects, anticancer effects, and regulation of bone metabolism (Du et al., 2024).

The total saturated fatty acid (SFA) content ranged from 5.54 % to 6.14 % across all samples. The primary SFAs were palmitic acid (3.15–3.60 %) and stearic acid (2.37–2.53 %). The PUFA/SFA ratio suggests that oils obtained via SBE and SPE are of higher-quality than those from conventional methods. The extraction method plays a crucial role in determining the physicochemical properties and fatty acid profiles of FSTOs, highlighting their potential for further development.

### 3.3 Characterization of bioactive compounds of oils extracted using various methods

Tocopherols, phytosterols, and polyphenols are essential natural antioxidants in vegetable oils that enhance oxidative stability and provide health benefits. This study demonstrates that different extraction methods result in varying concentrations of tocopherols, phytosterols, and polyphenols, as shown in Table 2.

**Table 2:** Bioactive compounds, oxidative stability, and Arrhenius model parameters of FSTOs extracted with different methods.

Chemical component	Samples			
	CP	SE	SBE	SPE
Tocopherol (μg/g)				
α-tocopherol	264.22 ± 6.67^a^	186.18 ± 11.32^c^	229.61 ± 2.09^b^	106.78 ± 9.39^d^
β-tocopherol	444.28 ± 13.74^b^	480.40 ± 26.48^a^	476.59 ± 6.22^a^	349.27 ± 8.12^c^
γ and δ-tocopherol	nd	nd	nd	nd
Sum	708.50 ± 20.33^a^	666.58 ± 37.39^b^	706.20 ± 6.84^a^	456.05 ± 16.55^c^

Phytosterol (mg/100 g)				
Glutinol	105.85 ± 17.52^a^	45.26 ± 6.93^b^	43.16 ± 5.65^b^	37.04 ± 0.16^b^
Stigmasterol	103.58 ± 21.48^a^	69.36 ± 12.04^ab^	55.49 ± 5.23^ab^	29.33 ± 9.82^b^
β-Sitosterol	140.91 ± 1.55^a^	134.45 ± 0.60^b^	137.67 ± 3.61^ab^	127.78 ± 0.34^c^
Δ7-Stigmastenol	7.09 ± 0.06^ab^	5.36 ± 0.91^b^	7.47 ± 1.06^ab^	7.66 ± 0.70^a^
β-Amyrin	20.09 ± 0.46^ab^	17.89 ± 2.13^b^	22.54 ± 0.13^ab^	23.91 ± 2.99^a^
Δ7-Avenasterol	19.36 ± 0.49^a^	13.21 ± 5.39^a^	16.54 ± 3.23^a^	20.13 ± 6.63^a^
Cycloartenol	28.79 ± 1.05^a^	24.94 ± 1.47^bc^	27.93 ± 0.27^ab^	23.33 ± 1.61^c^
Thunbergol	3.04 ± 0.53^a^	3.17 ± 0.48^a^	3.81 ± 0.98^a^	4.06 ± 0.72^a^
24-Methylene cycloartanol	6.02 ± 0.26^ab^	4.68 ± 1.12^b^	7.31 ± 0.41^a^	6.43 ± 0.58^ab^
Sum	434.72 ± 35.53^a^	318.31 ± 24.65^b^	321.92 ± 3.61^b^	279.67 ± 22.55^b^
TPC (μg GAE/g)	58.26 ± 1.05^b^	49.44 ± 6.40^c^	62.08 ± 0.60^a^	60.39 ± 1.12^a^
TFC (μg RE/g)	115.156 ± 2.091^a^	112.152 ± 1.033^b^	113.501 ± 0.618^ab^	110.136 ± 1.027^c^
OSI (h)	0.86 ± 0.03^b^	1.07 ± 0.04^ab^	1.24 ± 0.14^a^	1.07 ± 0.18^ab^

Arrhenius model parameters				
*E*_a_ (KJ/mol)	26.1444 ± 0.4329	26.0243 ± 0.4688	25.9562 ± 0.4228	25.8044 ± 0.4293
A (Pa s)	3.2726E-6 ± 5.9618E-7	3.4176E-6 ± 6.7399E-7	3.3959E-6 ± 6.0379E-7	3.3757E-6 ± 6.0924E-7
*R*^2^	0.9955	0.9947	0.9956	0.9954

*Note*: nd: not detected; TPC: total phenols content; GAE: gallic acid equivalent; TFC: total flavonoids content; RE: rutin equivalents; OSI: oxidation stability index; Different letters indicate significant differences at *P* < 0.05.

Tocopherols, important natural antioxidants in vegetable oils, possess anti-proliferative and anti-inflammatory properties and improve oxidative stability by inhibiting PUFA oxidation (Qin et al., 2024). In this study, α- and β-tocopherols were identified based on their HPLC retention times. β-Tocopherol exhibited the highest concentration among all oils; SE and SBE oils containing 480.40 μg/g and 476.59 μg/g, respectively, significantly exceeding levels obtained through other methods (*p* < 0.05). Conversely, α-Tocopherol exhibited the highest concentration in CP oil (264.22 μg/g), significantly higher than those in SE (186.18 μg/g), SBE (229.61 μg/g), and SPE (106.78 μg/g) oils. The total tocopherol content showed no significant difference (*p* > 0.05) between CP oil (708.50 μg/g) and SBE oil (706.20 μg/g). In contrast, SE and SPE oils contained significantly lower total tocopherols (666.58 μg/g and 456.05 μg/g, respectively) compared to CP and SBE oils (*p* < 0.05), suggesting that CP and SBE methods better preserve these antioxidants. The absence of γ- and δ-tocopherols across all samples indicates a specific composition favoring the retention of α- and β-tocopherols as principal antioxidants.

Table 2 illustrates significant variations in individual and total phytosterol contents among FSTOs extracted using different methods. CP oil exhibited the highest total phytosterol content (434.72 mg/100 g), significantly exceeding that of SE (318.31 mg/100 g), SBE (321.92 mg/100 g), and SPE (279.67 mg/100 g) oils. The predominant phytosterol, β-sitosterol, was notably concentrated in CP oil (140.91 mg/100 g), whereas SE, SBE, and SPE oils contained 134.45 mg/100 g, 137.67 mg/100 g, and 127.78 mg/100 g, respectively. Glutinol (37.04–105.85 mg/100 g) and stigmasterol (29.33–103.58 mg/100 g) together constituted 23.73–48.18 % of the total phytosterol content. Six additional phytosterols were identified, each present at significantly lower concentrations than β-sitosterol, glutinol, and stigmasterol. These phytosterols include Δ7-stigmastenol (5.36–7.66 mg/100 g), β-amyrin (17.89–23.91 mg/100 g), Δ7-avenasterol (13.21–20.13 mg/100 g), cycloartenol (23.33–28.79 mg/100 g), thunbergol (3.04–4.06 mg/100 g), and 24-methylene cycloartanol (4.68–7.31 mg/100 g). Phytosterols play a crucial role in lowering cholesterol levels and possess antioxidant, anti-inflammatory, and antibacterial properties. They also regulate thyroid function and promote cell growth. These benefits indicate that FSTOs have considerable potential for health-promoting applications. This finding is consistent with previous studies highlighting the significance extraction methods in enhancing the nutritional value of oils (Delfan-Hosseini et al., 2017; Hou et al., 2024; Liu et al., 2022), reinforcing the role of FSTOs as a dietary source of phytosterols.

Phenolic compounds and flavonoids are critical for oxidative stability and provide health benefits due to their antioxidant properties (Zamora & Hidalgo, 2016). As shown in Table 2, the TPC of oils extracted using different methods varied significantly (*p* < 0.05), ranging from 49.44 to 62.08 μg GAE/g. Notably, SBE oil exhibited the highest TPC at 62.08 μg GAE/g, suggesting that SBE is more effective in extracting phenolic compounds. The TFC ranged from 110.136 to 115.156 μg RE/g (Table 2), indicating significant variations among the samples (*p* < 0.05). Notably, CP and SBE oils exhibited the highest TFC values (115.156 μg RE/g and 113.501 μg RE/g, respectively), with no significant difference between them, suggesting that SBE effectively retains flavonoids at a level comparable to CP. In contrast, SPE oil recorded the lowest TFC (110.136 μg RE/g), likely due to the high pressure and temperature conditions that may promote flavonoid degradation (Liu et al., 2023). SE oil exhibited a slightly lower TFC (112.152 μg RE/g) than SBE, highlighting differences in solvent interactions with flavonoid compounds.

These bioactive compounds contribute not only to the nutritional quality of the oil, but also to its functional properties. Functional oils are generally defined as edible oils enriched with health-promoting constituents such as PUFAs, tocopherols, phytosterols, and polyphenols, which exert antioxidant, anti-inflammatory, and lipid-lowering effects (Farag et al., 2021). Therefore, the enrichment of these compounds in SBE and CP oils supports their potential application as functional oils in nutraceuticals or health-oriented food products.

### 3.4 Oxidative stability of oils extracted using various methods

Oxidative stability is vital for assessing vegetable oil quality, significantly affecting shelf life and culinary suitability. In this study, oils extracted by different methods showed significant variations in their oxidative stability index (OSI) (Table 2). SBE oil exhibited the highest OSI value (1.24 h), significantly exceeding CP oil (0.86 h) and SE and SPE oils (1.07 h each). These findings are consistent previous studies indicating that extraction methods significantly influence antioxidant content and oxidative stability (Gu et al., 2019; Liu et al., 2022; Thilakarathna et al., 2023). The elevated OSI of SBE oil underscores the importance of extraction methods that preserve natural antioxidants, thereby enhancing shelf life and quality (Gu et al., 2019).

### 3.5 Rheological behaviors of oils extracted using various methods

**Fig. 1:**
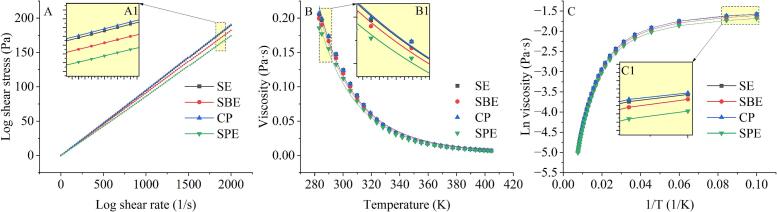
Flow behaviors (A), temperature-viscosity curves (B), and Arrhenius plots of ln viscosity versus 1/T (C) of FSTOs extracted using different methods. Insets (A1, B1, C1) show enlarged views of the corresponding curves.

As shown in Fig. 1A, all four oils displayed similar flow patterns at 25 °C. Shear stress was directly proportional to shear rate, confirming the Newtonian flow behavior characteristic of oils with long-chain molecules. Viscosities derived from flow curve slopes were 95.44, 94.73, 91.19, and 87.07 mPa·s for CP, SE, SBE, and SPE oils, respectively. The linear shear stress-shear rate relationship corroborates Newtonian flow behavior, aligning with our previous findings (Gu et al., 2019). Viscosity differences can be partially attributed to variations in the FA composition of the oils, as different extraction methods selectively extract varying proportions of fatty acids. Oils with higher MUFAs content, such as CP and SE oils, generally exhibit higher viscosities due to the more tightly packed molecular structure of MUFAs. Conversely, oils with a higher proportion of PUFAs, such as SBE and SPE oils, tend to exhibit lower viscosities due to the looser packing of the FA molecules and their capacity to reduce intermolecular friction (Delfan-Hosseini et al., 2017). Oils with lower free fatty acid content exhibit lower viscosities, as less shear force is required to overcome intermolecular forces during flow. This is corroborated by the acid values presented in Table 2.

The viscosity of FSTOs was analyzed over temperatures ranging from 10 to 130 °C. Consistent with typical plant oils behavior, all samples exhibited decreased viscosity with increasing temperature (Fig. 1B). This trend aligns with the theory that elevated temperatures enhance molecular motion, diminish intermolecular interactions, and weaken oil aggregates, thereby reducing viscosity (Zhu et al., 2024). Among the oils, SBE and SPE exhibited the lowest viscosities at all temperatures, consistent with steady shear measurement results. This suggests that the SBE and SPE methods yield less viscous oils, possibly due to the extraction processes affecting their molecular structure and composition. To further investigate viscosity's temperature dependence, Arrhenius plots (ln(viscosity) vs. 1/T) (Fig. 1C) were employed to determine the oils' thermodynamic parameters (Table 2). All oils exhibited *R*^2^ values exceeding 0.99, indicating adherence to an Arrhenius model and confirming their viscosity follow a temperature-dependent exponential relationship. The oils' activation energy (*E*_a_) values ranged from 25.8044 to 26.1444 kJ/mol. Notably, SBE and SPE oils exhibited the lowest *E*_a_ values, suggesting reduced sensitive to temperature variations compared to the other oils. These lower *E*_a_ values may be attributed to higher MUFA content and a more loosely packed molecular structure, facilitating easier flow at higher temperatures (Amakhmakh et al., 2024). This study confirms that extraction method influence the rheological behavior of the plant oils.

### 3.6 In vitro antioxidant activity of oils extracted using various methods

**Fig. 2:**
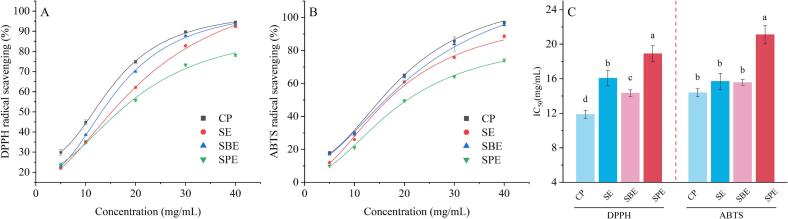
Fitted curves of DPPH (A) and ABTS (B) radical scavenging rates, and IC_50_ values (C) of FSTOs extracted using different methods.

Fig. 2A illustrates the fitted scavenging curve of DPPH radicals by FSTOs extracted using various methods. All oils exhibited increased antioxidant activity with rising concentrations. Notably, CP and SBE oils consistently exhibited higher DPPH radical scavenging capacities than SE and SPE oils. At 40 mg/mL, CP oil achieved the highest scavenging rate (94.58 %), followed by SBE oil at 93.36 %. Conversely, SE and SPE oils exhibited lower scavenging rates of 92.43 % and 78.17 %, respectively. Fig. 2C presents the IC_50_ values, further corroborating this trend. CP oil had the lowest IC_50_ value (11.857 mg/mL), followed by SBE oil (14.320 mg/mL), indicating that lower concentrations are required for 50 % scavenging. In comparison, SE and SPE oils exhibited higher IC_50_ values (16.062 mg/mL and 18.886 mg/mL, respectively), suggesting weaker antioxidant activity.

The ABTS radical scavenging assay showed similar fitted trends (Fig. 2B), with CP and SBE oils displaying superior antioxidant properties compared to SE and SPE oils. At 40 mg/mL, CP oil displayed the highest scavenging capacity (97.015 %), followed by SBE oil (95.51 %). SE and SPE oils showed lower values of 88.60 % and 73.90 %, respectively. The IC_50_ values for ABTS radical scavenging activity mirrored these trends (Fig. 2C). CP oil had the lowest IC_50_ value (14.382 mg/mL), followed by SBE oil (15.565 mg/mL). Conversely, SE and SPE oils exhibited higher IC_50_ values (15.696 mg/mL and 21.097 mg/mL, respectively).

The enhanced antioxidant activities in CP and SBE oils are attributed to their superior retention of bioactive compounds, such as tocopherols, phytosterols, and phenols. The differences in scavenging activity between CP/SBE oils and SE/SPE oils can be explained by the milder extraction conditions of CP and SBE, which likely preserve these bioactive compounds more effectively (see Table 2). It is important to distinguish between oxidative stability and antioxidant activity. Oxidative stability describes the oil matrix's resistance to oxidative degradation during storage or processing, whereas antioxidant activity refers to the ability of specific bioactive compounds (e.g., tocopherols, polyphenols, phytosterols) to scavenge free radicals and inhibit oxidation reactions.

### 3.7 VOCs analysis

#### 3.7.1 GC-IMS topographic plots

**Fig. 3:**
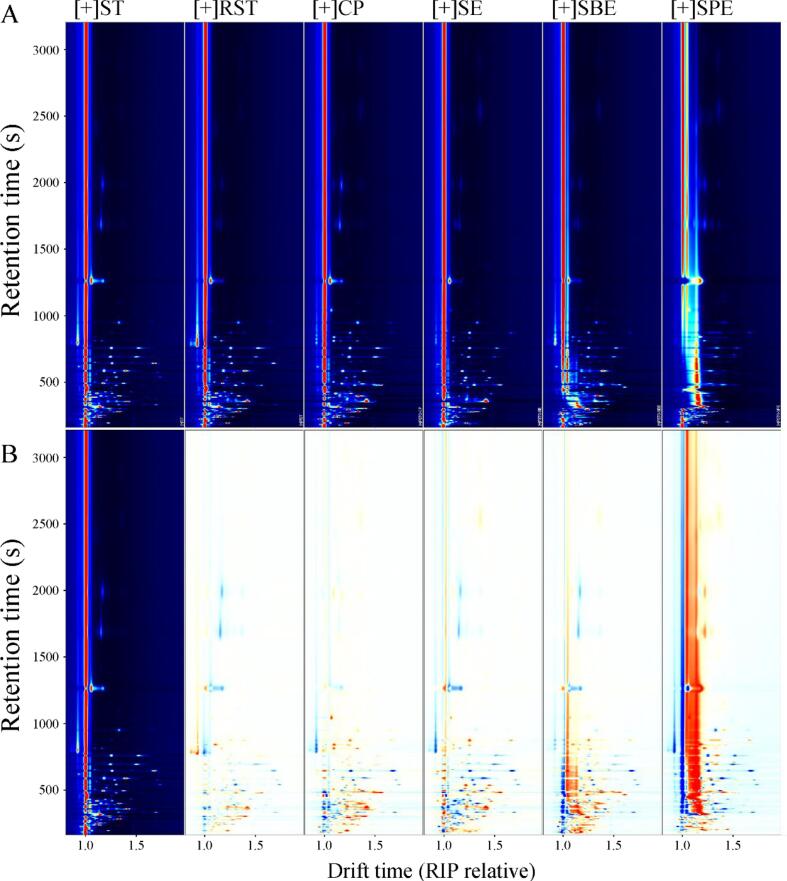
Top view (A) and difference view (B) of GC–IMS topographic plots of VOCs in seed and oil samples.

GC-IMS was employed for the first time to analyze the volatile profiles of seed and oil samples. Ion migration time and reactive ion peak (RIP) positions were normalized. The top view of the GC-IMS 3D topographic plot of six samples (see Fig. S1) is shown in Fig. 3A. Each point to the right of the RIP represents a volatile compound detected in the samples. A comparative model was used to analyze differences among the samples. Using ST as the reference, its signal peaks were subtracted from those of other spectra to highlight differences. Blue areas indicate substances at lower concentrations than in ST, while red areas indicate higher concentrations. The darker the color, the greater the difference (see Fig. 3B). The majority of VOCs were concentrated within a retention time of 100–600 s and a drift time of 1.0–1.6 ms. Significant differences were observed in the VOC profiles between ST and RST samples. RST exhibited increased concentration and variety of volatile compounds, likely due to Maillard reactions and thermal degradation of seed components, which form desirable aroma-active compounds (Zhang et al., 2021). This increase in VOCs in RST underscores the impact of roasting on enhancing the aromatic properties of the seeds. When comparing the VOC profiles of oils extracted using different methods, CP oil retained many volatile compounds found in RST, suggesting that cold pressing effectively captures natural aromas. However, solvent-based extraction methods (SE, SBE, and SPE) exhibited distinct differences in both the types and intensities of VOCs, likely due to each solvent's selective extraction capabilities and varying extraction conditions. Additionally, clear differences among SE, SBE, and SPE oils indicate that each extraction method uniquely influences the VOC composition, potentially altering the aroma and quality of the extracted oil. These results highlight the combined influence of roasting and extraction techniques on the VOC composition in seed oils, playing a critical role in the final product's flavor profile.

#### 3.7.2 GC-IMS fingerprint spectra and VOCs

**Fig. 4:**
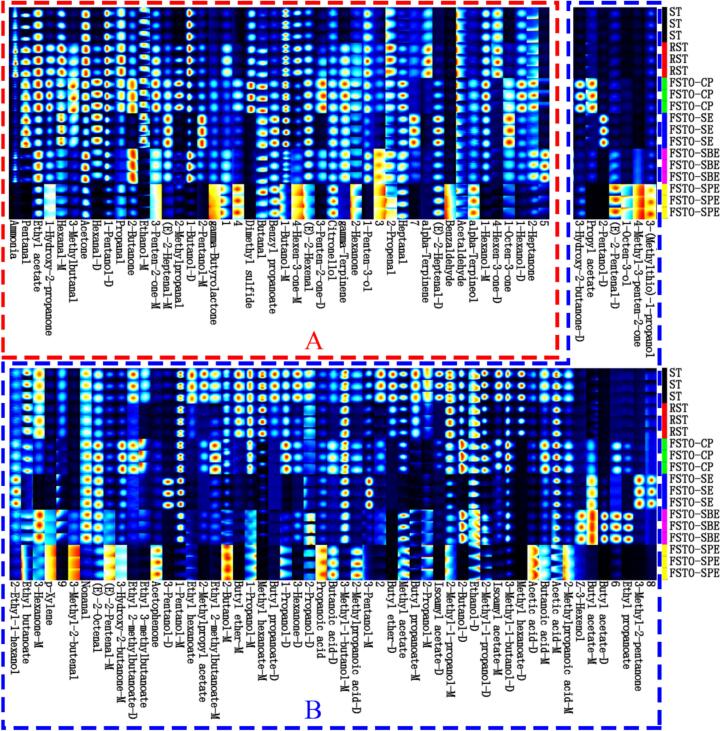
Fingerprint spectrum of VOCs in seed and oil samples. (M) and (D) denote monomer and dimer, respectively.

The Gallery Plot plugin enabled a comparative analysis of fingerprint spectra, highlighting differences in VOC composition across samples (Fig. 4). Each row represents all signals from a sample, while each column corresponds to signals of a specific VOC. Columns were ranked in descending order based on peak volume differences (RST - ST); redder areas indicate higher VOC signal intensity. Regions A and B denote VOCs exhibiting contrasting concentration changes due to roasting. Fig. 4A highlights VOCs with positive peak volume differences (RST - ST), indicating increased concentrations of these compounds after roasting. Conversely, Fig. 4B represents VOCs with negative peak volume differences (RST - ST), signifying decreased concentrations of these compounds due to roasting. As shown in Fig. 4 and Table 3, a total of 108 AOCs were identified, comprising 28 alcohols, 19 aldehydes, 19 esters, 17 ketones, 7 carboxylic acids, 3 terpenes, 2 ethers, 1 aromatic hydrocarbon, 1 lactone, 2 others, and 9 unidentified compounds. Notably, some compounds exhibited two peaks, corresponding to monomers and dimers. This phenomenon likely arises from compounds with high proton affinity, forming dimers as they traverse the drift tube (Xu, Wang, et al., 2023).

**Table 3:** The VOC compositions and integral parameters of five seed and oil samples based on HS-GC–IMS.

IMS code	VOCs	CAS	Formular	MW	RI	Rt (s)	Dt(RIPrel)	Peak volume (a.u.)					
								ST	RST	CP	SE	SBE	SPE
Alcohols (28)													
1	Citronellol	C106229	C10H20O	156.3	1885.2	2879.064	1.36198	913.75 ± 113.19^d^	1026.61 ± 128.06^d^	1425.17 ± 56.97^bc^	1513.91 ± 89.55^b^	1355.75 ± 29.44^c^	1942.25 ± 116.03^a^
4	3-(Methylthio)-1-propanol	C505102	C4H10OS	106.2	1801.5	2403.155	1.10423	1295.92 ± 61.4^b^	1285.78 ± 76.68^b^	1179.44 ± 68.75^c^	1226.82 ± 26.46^bc^	1303.45 ± 54.82^b^	2353.56 ± 50.47^a^
12	2-Ethyl-1-hexanol	C104767	C8H18O	130.2	1540.3	1366.484	1.42761	207.33 ± 34.83^d^	196.03 ± 28.39^d^	317.99 ± 16.31^b^	488.68 ± 39.85^a^	254.63 ± 13.77^c^	193.83 ± 10.08^d^
15	1-Octen-3-ol	C3391864	C8H16O	128.2	1488.3	1221.289	1.16262	63.11 ± 11.35^d^	57.68 ± 10.96^d^	147.18 ± 5.37^b^	109.37 ± 2.23^c^	132.54 ± 5.32^bc^	657.63 ± 52.89^a^
18	1-Hexanol-M	C111273	C6H14O	102.2	1372.7	951.478	1.33145	1692.8 ± 4.84^c^	1743.35 ± 14.81^b^	2410.57 ± 27.69^a^	1527.54 ± 13.07^c^	1329.92 ± 7.77^d^	273.39 ± 16.21^e^
19	1-Hexanol-D	C111273	C6H14O	102.2	1372.9	951.758	1.65119	230.69 ± 9.39^c^	251.97 ± 2.53^b^	473.01 ± 3.06^a^	179.98 ± 6.4^d^	185.59 ± 4.4^d^	77.98 ± 6.64^e^
26	1-Pentanol-M	C71410	C5H12O	88.1	1268	761.331	1.25862	2977.09 ± 6.76^b^	2904.63 ± 58.63^c^	3171.64 ± 33.34^a^	2634.59 ± 15.11^d^	2276.48 ± 20.14^e^	634.27 ± 19.73f
27	1-Pentanol-D	C71410	C5H12O	88.1	1267.8	761.06	1.52237	3027.2 ± 3.86^c^	3639.01 ± 15.84^a^	3416.43 ± 43.87^b^	1663.95 ± 9.67^e^	2118.63 ± 5.63^d^	1183.66 ± 30.72f
29	3-Methyl-1-butanol-M	C123513	C5H12O	88.1	1223.5	695.291	1.24616	2274.25 ± 3.41^b^	2009.09 ± 24.06^c^	2321.7 ± 0.69^a^	1753.59 ± 18.54^d^	1646.12 ± 11.22^e^	374.76 ± 10.5f
30	3-Methyl-1-butanol-D	C123513	C5H12O	88.1	1223.9	695.884	1.49054	2844.75 ± 6.22^b^	1571.5 ± 87.61	3147.98 ± 21.1^a^	904.7 ± 9.25	1735.15 ± 5.66^c^	680.03 ± 16.71
36	1-Penten-3-ol	C616251	C5H10O	86.1	1177.3	620.042	0.93607	607.75 ± 1.59^bc^	691.96 ± 87.17^a^	575.55 ± 12.04^c^	264.38 ± 18.45^d^	658.78 ± 27.97^ab^	129.96 ± 13.14^e^
37	1-Butanol-M	C71363	C4H10O	74.1	1160.6	585.677	1.18558	3171.74 ± 27.95^b^	3333.38 ± 21.41^a^	3292.73 ± 4.01^a^	2648.2 ± 30.96^c^	2543.54 ± 81.51^c^	3177.64 ± 129.5^b^
38	1-Butanol-D	C71363	C4H10O	74.1	1160.6	585.677	1.39197	4567.5 ± 5.03^c^	4803.75 ± 16.47^b^	2847.35 ± 15.04^d^	1072.57 ± 9.71^e^	5423.47 ± 42.44^a^	653.55 ± 16.8f
46	3-Pentanol-M	C584021	C5H12O	88.1	1124.7	517.78	1.20893	854.12 ± 4.8^b^	526.97 ± 16.85^d^	462.21 ± 10.15^e^	1604.91 ± 25.1^a^	416.82 ± 7.49f	596.07 ± 55.44^c^
47	3-Pentanol-D	C584021	C5H12O	88.1	1125	518.349	1.41948	97.81 ± 1.46^b^	39.06 ± 3.65^d^	47.31 ± 3.6^c^	596.87 ± 0.43^a^	24.13 ± 3.38^e^	40.06 ± 3.56^d^
50	2-Methyl-1-propanol-M	C78831	C4H10O	74.1	1111.8	495.377	1.1761	2755.49 ± 3.95^a^	2242.81 ± 49.91^b^	1554.54 ± 9.62^d^	694.76 ± 10.68f	1025.69 ± 21.34^e^	1696.98 ± 63.98^c^
51	2-Methyl-1-propanol-D	C78831	C4H10O	74.1	1112	495.71	1.37462	2184.39 ± 3.36^b^	1031.19 ± 73.16^d^	2315.09 ± 20.73^a^	359.32 ± 5.37^e^	2085.21 ± 114.14^c^	947.8 ± 27.98^d^
55	2-Pentanol-M	C6032297	C5H12O	88.1	1136.4	538.99	1.22096	341.5 ± 2^d^	568.38 ± 52.04^c^	686.32 ± 2.82^b^	2042.25 ± 11.85^a^	700.74 ± 2.64^b^	139.14 ± 14.15^e^
62	1-Propanol-M	C71238	C3H8O	60.1	1057.3	419.934	1.11357	2806.71 ± 9.89^a^	2618.38 ± 44.19^b^	2678.55 ± 32.87^b^	418.08 ± 11.83^e^	1260.4 ± 154.03^d^	2387.31 ± 63.02^c^
63	1-Propanol-D	C71238	C3H8O	60.1	1057.6	420.355	1.25894	1013.21 ± 6.63^b^	808.49 ± 48.94^c^	1414.33 ± 13.72^a^	144.8 ± 15.05^e^	375.91 ± 1.22^d^	1025.8 ± 70.38^b^
64	2-Butanol-M	C78922	C4H10O	74.1	1040.9	400.6	1.15352	1939.31 ± 21.53^b^	1778.91 ± 5.96^c^	1366.72 ± 39.84^d^	655.51 ± 41.81f	1174.87 ± 29.52^e^	2329.45 ± 217.95^a^
65	2-Butanol-D	C78922	C4H10O	74.1	1040.9	400.6	1.32774	2783.45 ± 6.88^b^	2206.75 ± 162.82^c^	6290.99 ± 34.22^a^	237.94 ± 17.09^d^	6243.69 ± 79.01^a^	208.9 ± 7.19^d^
72	Ethanol-M	C64175	C2H6O	46.1	946.3	315.696	1.04699	2760 ± 46.9^e^	3124.46 ± 25^d^	3593.53 ± 37.13^c^	4262.07 ± 18.09^b^	7808.27 ± 107.16^a^	1866.61 ± 35.02f
73	Ethanol-D	C64175	C2H6O	46.1	946.9	316.116	1.13244	7924.28 ± 21.76^c^	7327.68 ± 14.55^d^	5218.62 ± 15.64^e^	4369.93 ± 36.39f	41,385.01 ± 334.94^b^	65,537.98 ± 804.84^a^
74	2-Propanol-M	C67630	C3H8O	60.1	932.7	306.449	1.09582	537.97 ± 8.29^a^	165.73 ± 5.34^d^	43.32 ± 3.16f	85.87 ± 2.34^e^	233.19 ± 42.33^c^	355.65 ± 27.87^b^
75	2-Propanol-D	C67630	C3H8O	60.1	933.9	307.29	1.22787	1489.18 ± 14.72^b^	1274.82 ± 8.33^c^	779.9 ± 20.62^e^	641.35 ± 10.28f	1636.62 ± 75.18^a^	887.35 ± 110.67^d^
97	Z-3-Hexenol	C928961	C6H12O	100.2	1418.8	1050.974	1.24156	ND	ND	ND	ND	241.8 ± 4.06	ND
105	2-Pentanol-D	C6032297	C5H12O	88.1	1136.4	538.943	1.45511	92.68 ± 6.59^c^	89.41 ± 11.01^c^	126.8 ± 3.31^b^	1347.02 ± 11.04^a^	59.04 ± 1.27^d^	26.23 ± 1.26^e^

Aldehydes (19)													
11	Benzaldehyde	C100527	C7H6O	106.1	1552.9	1404.212	1.15256	235.3 ± 15.23^d^	293.56 ± 29.37^c^	315.96 ± 9.08^c^	239.21 ± 18.35^d^	357.9 ± 7.55^b^	3133.33 ± 51.59^a^
16	(E)-2-Octenal	C2548870	C8H14O	126.2	1441.3	1103.532	1.33928	203.36 ± 4.95^c^	160.31 ± 9.97^d^	257.43 ± 3.67^a^	238.5 ± 9.93^b^	230.46 ± 7.56^b^	144.85 ± 18.71^d^
17	Nonanal	C124196	C9H18O	142.2	1406.5	1023.503	1.49694	152.54 ± 4.99^c^	116.4 ± 9.95^d^	163.35 ± 8.35^c^	177.71 ± 7.95^b^	193.71 ± 11.29^a^	79.47 ± 5.12^e^
20	(E)-2-Heptenal-M	C18829555	C7H12O	112.2	1333.4	874.033	1.25754	284.66 ± 2.59f	585.03 ± 10.11^d^	1279.78 ± 40.51^b^	1834.39 ± 44.54^a^	1194.45 ± 12.26^c^	465.62 ± 20.07^e^
28	(E)-2-Hexenal	C6728263	C6H10O	98.1	1233.8	710.104	1.1825	234.82 ± 6.3f	370.92 ± 4.64^e^	466.39 ± 11.06^c^	489.15 ± 4.68^b^	421.12 ± 7.7^d^	536.47 ± 25.95^a^
32	3-Methyl-2-butenal	C107868	C5H8O	84.1	1216.7	685.811	1.0942	76.5 ± 1.78^c^	54.2 ± 1.23f	66.36 ± 1.74^d^	65.54 ± 5.03^e^	186.55 ± 7.73^b^	321.12 ± 5.57^a^
41	(E)-2-Pentenal-M	C1576870	C5H8O	84.1	1150.4	565.532	1.10755	319.55 ± 5.39^b^	271.44 ± 28.38^c^	316.47 ± 13.16^b^	257.01 ± 2.41^cd^	235.16 ± 25.78^d^	527.96 ± 2.88^a^
42	(E)-2-Pentenal-D	C1576870	C5H8O	84.1	1150.7	566.124	1.36116	31.7 ± 2.46^d^	26.31 ± 6.5^de^	46.2 ± 1.88^c^	24.01 ± 2.62^e^	61.88 ± 4.87^b^	250.24 ± 5.91^a^
52	Hexanal-M	C66251	C6H12O	100.2	1102.4	479.73	1.30508	938.49 ± 2.05f	1961.98 ± 31.59^b^	1681.93 ± 30.63^c^	2057.18 ± 18.77^a^	1106.69 ± 39.57^e^	1216.99 ± 46.09^d^
53	Hexanal-D	C66251	C6H12O	100.2	1102.8	480.396	1.56528	139.35 ± 2.19^e^	874.12 ± 33.43^d^	2413.07 ± 13.31^a^	2355.88 ± 9.43^b^	1279.7 ± 35.23^c^	130.76 ± 0.52^e^
69	Pentanal	C110623	C5H10O	86.1	1002.9	358.989	1.42206	3137.64 ± 10.95^d^	6919.39 ± 45.68^c^	8807.09 ± 140.15^b^	10,030.8 ± 91.06^a^	1839.57 ± 41.24^e^	242.59 ± 17.58f
76	3-Methylbutanal	C590863	C5H10O	86.1	930.8	305.188	1.40098	173.1 ± 2.92^e^	1169 ± 14.95^b^	1896.23 ± 89.84^a^	1228.41 ± 14.8^b^	458.47 ± 9.91^c^	428.24 ± 75.5^c^
79	Butanal	C123728	C4H8O	72.1	891.3	279.969	1.27781	477.66 ± 11.24^c^	647.61 ± 3.77^bc^	1006.33 ± 3.77^a^	957.95 ± 18.1^a^	694.46 ± 18.76^b^	943.58 ± 248.62^a^
82	Propanal	C123386	C3H6O	58.1	816	237.517	1.14464	1527.57 ± 5.88^c^	2101.9 ± 236.22^a^	1816.81 ± 17.25^b^	794.98 ± 17.59^e^	687.61 ± 19.12^e^	1172.35 ± 62.54^d^
84	Acetaldehyde	C75070	C2H4O	44.1	763.6	211.878	1.02702	3163.9 ± 26.8^a^	3216.64 ± 57.48^a^	3017.03 ± 66.9^a^	2274.8 ± 30.86^c^	2112.75 ± 111.91^c^	2616.59 ± 398.44^b^
85	2-Methylpropanal	C78842	C4H8O	72.1	830.3	245.083	1.28113	41 ± 0.74^e^	331.33 ± 39.72^b^	749.15 ± 11.99^a^	54.93 ± 2.8^e^	267.4 ± 17^c^	140.46 ± 45.71^d^
86	2-Propenal	C107028	C3H4O	56.1	878.7	272.403	1.06697	202.27 ± 7.71^bc^	280.64 ± 11.72^a^	178.84 ± 3.06^cd^	147.4 ± 3.68^d^	199.93 ± 3.55^bc^	215.03 ± 47.77^b^
93	(E)-2-Heptenal-D	C18829555	C7H12O	112.2	1334.1	875.273	1.67144	46.85 ± 10.84f	106.74 ± 7.2^e^	366.19 ± 6.83^d^	766.47 ± 30.38^a^	435.83 ± 4.27^c^	678.42 ± 9.33^b^
95	Heptanal	C111717	C7H14O	114.2	1197.9	659.938	1.36941	73.6 ± 1.81^e^	148.08 ± 1.79^d^	256.46 ± 6.16^a^	191.93 ± 3.53^c^	262.14 ± 7.17^a^	239.09 ± 1.89^b^

Esters (19)													
2	Benzyl propanoate	C122634	C10H12O2	164.2	1824.5	2525.442	1.36088	880.72 ± 67.1^e^	1049.57 ± 106.09^d^	1920.1 ± 83.37^c^	3090.11 ± 70.44^a^	2236.9 ± 12.31^b^	2988.14 ± 128.59^a^
31	Ethyl hexanoate	C123660	C8H16O2	144.2	1232.2	707.734	1.34782	140.56 ± 1.55^a^	38.45 ± 2.73^b^	ND	ND	ND	ND
34	Methyl hexanoate-M	C106707	C7H14O2	130.2	1189	645.52	1.26362	2276.91 ± 10.58^a^	2086.24 ± 24.7^b^	461.39 ± 12.06^c^	329.37 ± 6.14^d^	309.81 ± 17.81^d^	95.81 ± 4.04^e^
35	Methyl hexanoate-D	C106707	C7H14O2	130.2	1189.3	646.113	1.68666	3361.22 ± 8.66^b^	1894.71 ± 106.52^a^	ND	ND	ND	ND
39	Butyl propanoate-M	C590012	C7H14O2	130.2	1155.9	576.197	1.28826	949.22 ± 13.14^a^	602.59 ± 16.27^b^	ND	ND	ND	ND
40	Butyl propanoate-D	C590012	C7H14O2	130.2	1156.8	577.974	1.72054	356.2 ± 1.47^a^	162.04 ± 12.65^b^	ND	ND	ND	ND
44	Isoamyl acetate-M	C123922	C7H14O2	130.2	1138.3	542.424	1.30572	1532.55 ± 5.39^a^	286.53 ± 2.79^e^	1034.09 ± 12.8^b^	431.63 ± 4.31^d^	512.16 ± 4.47^c^	213.64 ± 1.99f
45	Isoamyl acetate-D	C123922	C7H14O2	130.2	1138.3	542.424	1.74621	538.51 ± 5.8^a^	33.66 ± 8.25^e^	164.64 ± 1.48^b^	100.1 ± 3.41^c^	73.15 ± 3.05^d^	37.03 ± 2.92^e^
57	Ethyl 3-methylbutanoate	C108645	C7H14O2	130.2	1083	452.299	1.2767	59.98 ± 3.47^b^	8.55 ± 1.9^e^	76.69 ± 1.57^a^	13.15 ± 0.29^d^	33.61 ± 1.64^c^	8.92 ± 0.96^e^
58	Ethyl 2-methylbutanoate-M	C7452791	C7H14O2	130.2	1070.2	435.906	1.25894	227.98 ± 5.41^b^	77.59 ± 28.83^c^	288.54 ± 1.18^a^	74.75 ± 3.47^c^	89.46 ± 3.92^c^	15.77 ± 0.28^d^
59	Ethyl 2-methylbutanoate-D	C7452791	C7H14O2	130.2	1069.9	435.486	1.64954	61.74 ± 2.25^b^	11.63 ± 0.81^d^	66.91 ± 1.6^a^	12.97 ± 2.49^d^	18.97 ± 0.31^c^	11.11 ± 0.92^d^
67	2-Methylpropyl acetate	C110190	C6H12O2	116.2	1031	389.251	1.60849	144.3 ± 1.5^a^	15.64 ± 3.43^d^	65.42 ± 0.11^b^	12.79 ± 0.95^de^	36.44 ± 6.23^c^	9.18 ± 0.13^e^
68	Ethyl butanoate	C105544	C6H12O2	116.2	1037.3	396.397	1.20679	668.58 ± 13.72^a^	654.05 ± 17.51^a^	463.51 ± 7.95^b^	236.03 ± 13.09^d^	307.56 ± 11.85^c^	ND
78	Ethyl acetate	C141786	C4H8O2	88.1	897.4	283.752	1.3344	3919.29 ± 48.63^d^	5348.43 ± 31.37^b^	5576.51 ± 27.64^a^	2734.52 ± 11.55^e^	4989.59 ± 22.02^c^	2579.17 ± 20.68f
80	Methyl acetate	C79209	C3H6O2	74.1	848.8	255.17	1.19125	931.16 ± 16.48^c^	588.79 ± 23.6^e^	684.52 ± 4.78^d^	91.55 ± 3.35f	1182.62 ± 15.26^b^	1427.41 ± 93.2^a^
98	Butyl acetate-M	C123864	C6H12O2	116.2	1093.4	466.115	1.23862	ND	ND	224.54 ± 3.93^c^	454.57 ± 19.38^a^	413.17 ± 37.52^b^	25.34 ± 1.09^d^
99	Butyl acetate-D	C123864	C6H12O2	116.2	1093.7	466.507	1.61501	ND	ND	52.19 ± 2.46^c^	162.49 ± 3.86^b^	1484.93 ± 2.37^a^	25.84 ± 4.56^d^
101	Ethyl propanoate	C105373	C5H10O2	102.1	975	336.122	1.44703	ND	ND	156.47 ± 1.25^b^	14.03 ± 1.56^c^	546.22 ± 2.8^a^	11.32 ± 2.97^c^
102	Propyl acetate	C109604	C5H10O2	102.1	995.8	351.784	1.47308	44.86 ± 0.66^c^	42.81 ± 2.35^cd^	306.01 ± 3.75^a^	39.86 ± 1.29^d^	70.62 ± 1.36^b^	6.77 ± 0.88^e^

Ketones (17)													
3	Acetophenone	C98862	C8H8O	120.2	1816.7	2483.249	1.18188	377.19 ± 75.87^cd^	324.83 ± 73.97^d^	539.35 ± 30.33^b^	482.33 ± 17.62^bc^	495.56 ± 13.26^bc^	2227.07 ± 154.05^a^
22	1-Hydroxy-2-propanone	C116096	C3H6O2	74.1	1317.1	843.72	1.09315	300.6 ± 4.31f	1532.42 ± 45.3^b^	1766.6 ± 21.64^a^	761.89 ± 9.28^e^	1069.03 ± 15.4^c^	928.16 ± 22.38^d^
23	3-Hydroxy-2-butanone-M	C513860	C4H8O2	88.1	1301.9	816.516	1.08991	457.23 ± 6.9^c^	407.29 ± 9.17^d^	1615.18 ± 31.3^a^	671.85 ± 10.48^b^	480.95 ± 8.17^c^	346.13 ± 14.81^e^
24	3-Hydroxy-2-butanone-D	C513860	C4H8O2	88.1	1301.5	815.739	1.33	45.61 ± 2.06^d^	44.45 ± 4.39^d^	679.63 ± 26.17^a^	109.56 ± 1.52^c^	95.56 ± 6.14^c^	221.86 ± 19.22^b^
33	4-Hexen-3-one-M	C2497214	C6H10O	98.1	1192.8	653.223	1.09317	491.43 ± 4.85^b^	652.91 ± 9.26^a^	289.47 ± 10.17^d^	260.76 ± 9.41^e^	296.6 ± 2.7^d^	454.23 ± 2.28^c^
43	4-Methyl-3-penten-2-one	C141797	C6H10O	98.1	1146.1	557.236	1.1363	111.73 ± 4.51^c^	103.38 ± 4.21^c^	53.65 ± 1.74^d^	42.38 ± 0.26^d^	392.4 ± 33.02^b^	824 ± 42.06^a^
49	3-Penten-2-one-M	C625332	C5H8O	84.1	1117.8	505.698	1.09646	413.67 ± 4.71^d^	750.66 ± 114.72^b^	1034.9 ± 11.27^a^	320.85 ± 4.42^e^	598.01 ± 17.48^c^	470.67 ± 9.52^d^
54	2-Hexanone	C591786	C6H12O	100.2	1100.6	476.733	1.19628	129.36 ± 5.47f	236.51 ± 7.92^c^	272.51 ± 3.58^b^	172.2 ± 4.95^d^	147.74 ± 7.25^e^	337.58 ± 18.27^a^
56	4-Hexen-3-one-D	C2497214	C6H10O	98.1	1192.8	653.21	1.45424	193.16 ± 0.56^b^	228.44 ± 6.4^a^	ND	ND	ND	ND
60	3-Hexanone-M	C589388	C6H12O	100.2	1066.9	431.703	1.19902	341.76 ± 24.15^b^	325.41 ± 55.72^b^	132.9 ± 3.98^c^	17.08 ± 0.37^d^	436.8 ± 13.55^a^	122.55 ± 2.26^c^
61	3-Hexanone-D	C589388	C6H12O	100.2	1067.2	432.124	1.45646	283.11 ± 2.66^a^	69.81 ± 35.7^d^	168.55 ± 3.69^b^	17.24 ± 0.88^e^	103.31 ± 0.83^c^	25.62 ± 0.4^e^
77	2-Butanone	C78933	C4H8O	72.1	916.7	295.941	1.24341	1042.21 ± 2.86^d^	1585.21 ± 105.69^c^	3815.94 ± 41.3^b^	267.05 ± 3.2^e^	4715.29 ± 88.78^a^	957.69 ± 21.25^d^
81	Acetone	C67641	C3H6O	58.1	837.3	248.866	1.11468	4571.38 ± 48.47^b^	5381.86 ± 69.47^a^	4327.64 ± 5.39^c^	2312.27 ± 22.3^e^	4613.16 ± 18.93^b^	2491.34 ± 75^d^
87	3-Penten-2-one-D	C625332	C5H8O	84.1	1118.3	506.52	1.3466	54.68 ± 1.49^c^	169.7 ± 52.83^b^	410.94 ± 4.8^a^	19.73 ± 1.43^c^	194.01 ± 2.01^b^	26.15 ± 0.42^c^
94	1-Octen-3-one	C4312996	C8H14O	126.2	1313	836.284	1.25959	15.58 ± 2.21f	48.94 ± 4.31^e^	169.61 ± 6.01^b^	247.06 ± 15.25^a^	127.27 ± 3.04^c^	90.21 ± 4.51^d^
104	3-Methyl-2-pentanone	C565617	C6H12O	100.2	1054.7	416.781	1.46949	ND	ND	66.88 ± 1.22^c^	610.38 ± 5.95^a^	83.56 ± 0.49^b^	22.73 ± 2.94^d^
108	2-Heptanone	C110430	C7H14O	114.2	1194.6	655.545	1.61944	43 ± 4.42^d^	53.7 ± 5.67^c^	88.07 ± 4.36^a^	32.11 ± 2.21^e^	79.63 ± 3.34^b^	41.26 ± 0.51^d^

Carboxylic Acids (7)													
5	Butanoic acid-M	C107926	C4H8O2	88.1	1709.2	1968.482	1.17433	3790.32 ± 67.18^b^	1284.45 ± 134.97^e^	4550.25 ± 207.62^a^	1798.95 ± 109.81^d^	1394.39 ± 43.54^e^	3334.51 ± 347.52^c^
6	Butanoic acid-D	C107926	C4H8O2	88.1	1710.6	1974.252	1.37345	451.39 ± 72.04^b^	197.71 ± 42.52^c^	700.64 ± 46.15^a^	265.76 ± 3.54^c^	238.82 ± 2.36^c^	725.45 ± 64.99^a^
7	2-Methylpropanoic acid-M	C79312	C4H8O2	88.1	1635.9	1679.983	1.15605	5533.98 ± 93.32^b^	1833.33 ± 212.16^e^	5199.8 ± 154.58^c^	2274.16 ± 72.01^d^	2240.87 ± 12.69^d^	7399.9 ± 284.02^a^
8	2-Methylpropanoic acid-D	C79312	C4H8O2	88.1	1637.4	1685.753	1.37142	443.01 ± 36.38^b^	136.4 ± 33.67^c^	477.18 ± 37.72^b^	190.87 ± 4.32^c^	168.8 ± 11.15^c^	770.9 ± 56.19^a^
9	Propanoic acid	C79094	C3H6O2	74.1	1639.5	1693.446	1.11744	1074.51 ± 41.4^c^	852.16 ± 57.43^d^	1274.54 ± 56.87^b^	1213.31 ± 15.42^b^	984.2 ± 90.82^c^	1425.31 ± 57.69^a^
13	Acetic acid-M	C64197	C2H4O2	60.1	1504.4	1264.733	1.05975	17,277.54 ± 18.35^a^	13,675.79 ± 76.07^d^	16,170.9 ± 16.3^b^	11,771.18 ± 214.1f	14,428.18 ± 80.28^c^	12,806.59 ± 594.02^e^
14	Acetic acid-D	C64197	C2H4O2	60.1	1504.4	1264.733	1.16709	3709.61 ± 12.52^b^	1606.29 ± 29.04^d^	2663.52 ± 16.92^c^	961.61 ± 46.61^e^	1476.24 ± 16.69^d^	15,351.85 ± 245.89^a^

Terpenes (3)													
89	α-Terpinene	C99865	C10H16	136.2	1193.5	654.05	1.22565	107.47 ± 3.38^b^	175.16 ± 4.54^a^	30.5 ± 1.32^c^	19.82 ± 0.19^e^	24.87 ± 1.48^d^	14.47 ± 1.22f
91	γ-Terpinene	C99854	C10H16	136.2	1250.5	734.624	1.22465	16.32 ± 0.54^e^	125.98 ± 9.81^c^	178.79 ± 5.54^a^	128.41 ± 4^bc^	136.76 ± 4.86^b^	30.28 ± 5.43^d^
92	α-Terpineol	C98555	C10H18O	154.3	1905.5	3008.581	1.22771	310.86 ± 73.75^d^	362.48 ± 63.95^d^	753.77 ± 90.92^b^	633.38 ± 12.01^c^	664.18 ± 26.62^bc^	1063.61 ± 11.93^a^

Ethers (2)													
70	Butyl ether-M	C142961	C8H18O	130.2	973.5	335.03	1.32996	853.09 ± 0.71^a^	675.03 ± 28.05^b^	ND	ND	ND	ND
71	Butyl ether-D	C142961	C8H18O	130.2	972.9	334.61	1.70281	668.35 ± 1.69^a^	330.53 ± 43.2^b^	ND	ND	ND	ND

Aromatic Hydrocarbon (1)													
48	p-Xylene	C106423	C8H10	106.2	1134.6	535.661	1.09534	118.83 ± 5.17^c^	102.01 ± 7.17^d^	119.72 ± 2.7^c^	58.09 ± 3.77^e^	211.01 ± 3.37^b^	363.05 ± 4.17^a^

Lactones (1)													
10	γ-Butyrolactone	C96480	C4H6O2	86.1	1711.9	1980.022	1.09204	515.3 ± 21.46^e^	739.56 ± 42.57f	1479.52 ± 22.4^a^	1052.43 ± 37.5^d^	1294.42 ± 9.19^b^	1106.49 ± 23.77^c^

Others (2)													
25	Ammonia	C7664417	H3N	17	1289.1	794.753	0.92552	17,468.18 ± 90.75^b^	26,720.29 ± 1662.42^a^	7695.86 ± 630.79^d^	6032.85 ± 135.01^e^	12,889.76 ± 381.92^c^	2599.59 ± 399.8f
83	Dimethyl sulfide	C75183	C2H6S	62.1	795.2	227.009	0.95822	1547.12 ± 9.76^c^	1737.84 ± 35.09^b^	2704.5 ± 7.61^a^	217.19 ± 3.16^e^	482.6 ± 10.06^d^	150.9 ± 18.89f

Unidentified (9)													
21	1	*	*	0	1335.5	877.919	1.38948	106.12 ± 4.76^d^	327.19 ± 33.55^b^	169.39 ± 20.95^c^	146.19 ± 8.35^cd^	292.38 ± 35.95^b^	413 ± 28.33^a^
66	2	*	*	0	1031	389.251	1.2889	390.12 ± 8.52^a^	62.48 ± 1.22^d^	122.05 ± 7.87^b^	44.46 ± 1.49^e^	103.51 ± 7.33^c^	49.76 ± 2.87^e^
88	3	*	*	0	1147.1	559.059	1.07806	102.46 ± 8.12^d^	183.43 ± 9.44^c^	193.41 ± 3.89^c^	87.07 ± 1.45^d^	270.58 ± 20.48^a^	243.79 ± 6.98^b^
90	4	*	*	0	1414.3	1040.959	1.07148	213.87 ± 3.1f	418.41 ± 10.52^e^	1766.33 ± 26.16^a^	821.55 ± 11.92^c^	658.44 ± 35.11^d^	1378.55 ± 9.36^b^
96	5	*	*	0	1297.9	809.474	1.27388	32.57 ± 5.52^e^	41.19 ± 3.13^e^	154.29 ± 6.26^b^	59.92 ± 1.03^d^	417.68 ± 18.31^a^	114.23 ± 1.68^c^
100	6	*	*	0	1055.6	417.955	1.55213	ND	ND	122.95 ± 3.43^b^	27.14 ± 1.69^c^	204.5 ± 5.44^a^	31.16 ± 0.92^c^
103	7	*	*	0	1055.3	417.564	0.94668	87.65 ± 3.49^d^	156.78 ± 4.37^c^	84.79 ± 3.2^d^	511.88 ± 4.96^a^	176.59 ± 8.26^b^	39.99 ± 2.65^e^
106	8	*	*	0	1186.3	639.57	0.94129	ND	ND	97.22 ± 14.76^b^	489.31 ± 4.35^a^	67.19 ± 1.73^c^	76.34 ± 2.60^c^
107	9	*	*	0	1089.3	460.531	1.25835	187.37 ± 4.08^ab^	167.04 ± 8.72^b^	61.26 ± 2.06^d^	86.41 ± 4.57^c^	205.06 ± 34.39^a^	22.22 ± 1.81^e^

ST: Semen Trichosanthis; RST: roasted Semen Trichosanthis; CP: cold pressing; SE: Solvent extraction; SBE: subcritical fluid extraction; SPE: supercritical fluid extraction; M: monomer; D: dimer; RI: retention index; Rt: retention time; Dt: drift time. Retention index was calculated refereed to the retention time of C4–C9 n-ketones under the same conditions. Different letters indicate significant differences at *p* < 0.05.

The results indicate that roasting significantly increased the concentration of pleasant aroma compounds (Fig. 4A) and simultaneously reduced off-flavor substances (Fig. 4B), thereby enhancing the sensory profile of the seed oils. Compounds such as pentanal and ethyl acetate, known for their green, fruity, and sweet aromas (Xi et al., 2024), increased significantly during roasting, imparting fresh and desirable notes. Maillard reaction products, such as 1-hydroxy-2-propanone and 3-methylbutanal, which impart caramel, chocolate, and nutty scents (Shakoor et al., 2022), increased significantly, enriching the roasted profile. Conversely, off-flavor compounds such as 2-methylpropanoic acid, acetic acid, and butanoic acid, associated with spicy, sour, and rancid flavors (Ivanova-Petropulos et al., 2015), decreased significantly, indicating the degradation or suppression of these undesirable volatiles during roasting. This dual effect of roasting—enhancing desirable sensory attributes and eliminating harsh odors—demonstrates its crucial role in improving aroma balance. However, ammonia and dimethyl sulfide, compounds linked to undesirable odors, increased in concentration after roasting, likely due to the thermal degradation of proteins and sulfur-containing compounds. During extraction, the concentrations of both compounds decreased, except for dimethyl sulfide in CP oil, which increased; the other oils (SE, SBE, and SPE) exhibited reduced levels of these undesirable compounds.

The VOC profiles of FSTOs varied significantly depending on the extraction method. CP oil exhibited the lowest VOC diversity, primarily containing compounds inherent to the seeds' aroma, such as aldehydes and alcohols. In contrast, SE, SBE, and SPE oils demonstrated notably higher VOC abundance and complexity, especially SBE and SPE oils. These two methods enriched low molecular-weight volatile compounds, likely due to their higher solvating power and milder conditions. These findings highlight the significant impact of extraction techniques on VOC retention and transfer efficiency in FSTOs, consistent with previous studies on seed oil volatiles (Zeng et al., 2024; Zhang, Yuan, et al., 2022). CP oil exhibited the least diverse VOC profile, dominated by aldehydes and alcohols inherent to the seeds' natural aroma. Key VOCs in CP oil, such as pentanal, 2-butano, ethyl acetate, ethanol, and acetone, contribute to green, grassy, fruity, sweet, and aromatic notes (Zhang, Chen, et al., 2022). These findings indicate that CP retains primary aroma compounds but is less effective at extracting secondary metabolites like ketones and esters, leading to a more straightforward aroma profile. In contrast, SE oil displayed a broader range of VOCs, including esters (e.g., benzyl propanoate and ethyl acetate) and ketones (e.g., acetone, 1-hydroxy-2-propanone, 3-hydroxy-2-butanone, and 3-methyl-2-pentanone), along with aldehydes and alcohols. These compounds impart fruity and sweet aromas (Xu, Bi, et al., 2023), enhancing the sensory complexity of SE oil. This increased diversity is attributed to the solvent's capacity to dissolve a wider range of compounds and thermally induced reactions during extraction that generate additional volatiles. SBE oil exhibited the highest enrichment of VOCs, particularly alcohols, esters, Ketones, and terpenes. The abundance of compounds such as ethanol, 2-butanol,1-butanol, ethyl acetate, 2-butanone, and acetone in SBE oil suggests that subcritical conditions effectively extract volatiles while minimizing thermal degradation (Liu et al., 2020). The mild extraction conditions likely preserve sensitive aroma compounds, contributing to the oil's characteristic floral and fruity notes (Alañón et al., 2017). SPE oil also exhibited a complex VOC profile, similar to SBE, with slightly higher levels of oxygenated compounds like ketones and alcohols. Acetic acid, 2-methylpropanoic acid, butanoic acid, and benzaldehyde exemplify the potent solvating ability of supercritical CO₂, particularly for polar and semi-volatile compounds (Ahangari et al., 2021). These compounds impart sweet and creamy aroma attributes (Ma et al., 2024), enriching the sensory profile of SPE oil.

Notably, several VOCs identified in FSTOs were absent from ST and RST samples. These VOCs, including Z-3-hexenol, butyl acetate, ethyl propanoate, and 3-methyl-2-pentanone, may form during extraction through esterification or other chemical reactions (Dong et al., 2024). Conversely, VOCs such as methyl hexanoate and butyl ether, found in ST and RST samples, were notably absent from FSTOs. This suggests that these compounds may volatilize or degrade during extraction, particularly under elevated temperatures or prolonged extraction times (Dong et al., 2024). Overall, extraction methods affect both the retention of seed-derived VOCs and the formation of new volatiles, thereby enriching the aroma profile of FSTOs.

GC-IMS is well known for its high sensitivity in volatile compound analysis. However, it has certain limitations that can affect its accuracy. In our study, nine compounds, labeled as “1–9” in the fingerprint plot, were unidentified due to an incomplete IMS database. This observation is consistent with previous research, which noted that IMS libraries may not cover all volatile compounds, resulting in identification challenges (Xi et al., 2024; Xu, Wang, et al., 2023). Moreover, matrix effects and the limited resolution of IMS can affect compound ionization and hinder the separation of compounds with similar ion mobility. To improve identification accuracy, we recommend expanding the IMS database, integrating GC-IMS with GC–MS for better compound identification, using higher-resolution IMS, and applying advanced data processing techniques, such as multivariate analysis, to address these challenges and obtain more reliable results.

### 3.8 Principal component analysis (PCA) and correlation analysis of the physicochemical properties of FSTOs

**Fig. 5:**
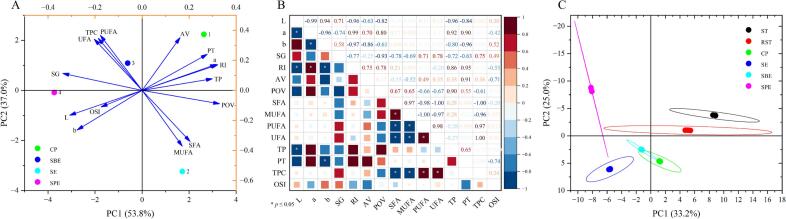
PCA and correlation analysis of the physicochemical properties of FSTOs (A & B). PCA of the VOCs of seed and FSTO samples (C).

PCA was conducted to systematically evaluate and compare the effects of different extraction methods on the physicochemical properties of FSTOs. As depicted in Fig. 5A, the PCA biplot reveals that the first principal component (PC1) accounting for 53.8 % of the total variance, while the second component (PC2) contributes 37.0 %, together capturing 90.8 % of the total variability. The PCA biplot distribution clear separates the oils by extraction method, highlighting the significant impact of these techniques on oil quality. SPE oil appears on the negative axis of PC1, indicating its low SG, RI, along with a high UFA content, especially PUFAs. This suggests that SPE's milder extraction conditions preserve more UFAs, enhancing oil quality regarding nutritional content and oxidative stability (Liu et al., 2023). Conversely, SE oil's positive position on PC1indicates higher SFA and MUFA levels, correlating with increased OSI. This aligns with the harsher solvent-based conditions of SE method, which may extract more thermally stable fatty acids such as SFAs and MUFAs, possibly reducing polyunsaturated compounds (Liu et al., 2022). Higher AV and POV values in SE oil might stem from extended solvent exposure and higher temperatures, which accelerate oxidation and degrade antioxidants (Liu et al., 2022). CP and SBE oils load mainly on PC2 but differ along PC1, indicating similar physicochemical properties yet varying in oxidative parameters and bioactive component preservation. CP oil, nearer to the origin on PC1, exhibits higher AV and POV values, possibly due to its mechanical extraction avoiding solvent use but failing to extract substantial antioxidative compounds (Thilakarathna et al., 2023). This may explain the moderate oxidation in CP oils, even though they retain some natural antioxidants. In contrast, SBE oil's position indicates lower oxidation levels, with moderate to high polyphenol and phytosterol content, likely due to the subcritical *n*-butane process uses lower temperatures and pressures than SE. This facilitates better phenolic and UFA preservation, improving oil quality (Hou et al., 2024). The biplot shows a positive correlation between TPC and MUFA, indicating that increased MUFA levels are associated with enhanced antioxidant capacity and oxidative stability.

Correlation analysis revealed significant relationships among physicochemical properties, fatty acid compositions, and bioactive components of the studied oils (Fig. 5B). The L* value negatively correlated with the a* value (*r* = −0.99), reflecting their inverse relationship. L* value also showed negative correlations with SG (*r* = −0.77) and AV (*r* = −0.96), indicating that as oil darkens (lower L* values), its density and acidity increase. This trend may result from oxidative processes leading to polymerization and pigmentation, which increase density and free fatty acid content (Farag et al., 2021). The b* value positively correlated with SG (*r* = 0.58) and RI (*r* = 0.75), suggesting that a more intense yellow hue corresponds to increased oil density and refractive power of the oil, possibly due to carotenoids and other pigments. SFA exhibited strong negative correlations with both SG (*r* = −0.97) and RI (*r* = −0.98), likely due to the linear structure of saturated fats leading to lower RI and density compared to unsaturated fatty acids. PUFA positively correlated with UFA (*r* = 0.98), as PUFAs are a subset of UFAs. PUFA also positively correlated with AV (*r* = 0.86) and negatively with OSI (*r* = −0.74), suggesting that oils richer in PUFA content exhibit higher acidity and reduced oxidative stability due to the susceptibility of polyunsaturated bonds to oxidation (Islam et al., 2023). MUFA showed a strong negative correlation with PUFA (*r* = −1.00), highlighting their reciprocal relationship in the oils. Higher MUFA levels, often linked to enhanced oxidative stability, negatively correlated with PUFA content, indicating that oils abundant in monounsaturated fats may possess longer shelf-life than those rich in polyunsaturated fats. OSI negatively correlated with UFA content (r = −0.96), indicating that oils high in unsaturated fatty acids, particularly PUFAs, are more susceptible to oxidation. Conversely, OSI positively correlated with TPC (*r* = 0.24), highlighting the protective effect of phenolic compounds on oxidative stability. Phenolic compounds are renowned antioxidants that inhibit UFA oxidation of, thereby prolonging oil stability (Machado et al., 2023). TPC positively correlated with MUFA content (*r* = 0.97) and TP content (*r* = 0.65), indicating that oils higher in MUFAs often contain more phenolic compounds, enhancing nutritional value and antioxidative capacity. The relationship between these bioactive components and oil stability underscores the need to preserve phenolic compounds during processing. The POV, a key indicator of lipid peroxidation, showed positive correlation with SG (*r* = 0.67), AV (*r* = 0.90), and PUFA content (*r* = 0.55). This suggests that oils with higher POVs, indicative of early-stage oxidation, tend to exhibit increased density, acidity, and PUFA levels, which can lead to oil degradation during storage and processing. TP content exhibited positive correlations with the b* value (r = 0.75) and POV (r = 0.55), suggesting that phytosterols may affect both the color and oxidative state of oils. The observed correlations among physicochemical properties, fatty acid profiles, and bioactive components underscore the importance of carefully selecting processing methods to optimize oil quality. Oils with higher UFA levels, particularly polyunsaturated types, are more prone to oxidative degradation. However, phenolic compounds and phytosterols can mitigate this susceptibility. Therefore, optimizing the extraction and preservation of these bioactive compounds is critical for enhancing oil stability and extending shelf-life.

### 3.9 PCA of the VOC profiles of seed and FSTO samples

PCA was conducted to assess differences in VOCs between seed and oil samples. The PCA plot (Fig. 5C) shows a clear separation between seed and oil samples, indicating distinct volatile profiles for each group. The first two principal components, PC1 and PC2, account for 33.2 % and 25.0 % of the variance, respectively, totaling a cumulative contribution of 58.2 %. This substantial contribution effectively reveals meaningful differences among the samples and differentiates the unique volatile characteristics of each extraction method.

In the PCA biplot, seed samples ST and RST are located in the upper right quadrant, forming a distinct cluster separate from the oil samples. The spatial separation along PC1 and PC2 reflects significant differences in VOC composition, suggesting that seeds may retain compounds that are lost or altered during oil extraction. This separation is consistent with previous findings that raw materials and extracted oils typically exhibit differing volatile profiles due to processing and extraction influences (Xi et al., 2024; Xu, Wang, et al., 2023).

Each extraction method forms a distinct cluster in the PCA plot, confirming the unique volatile profiles associated with each technique. SPE oil appears in the top left quadrant, distinctly separated, indicating that SPE produces unique volatiles influenced by its low-temperature, high-pressure conditions. This finding aligns with studies reporting minimal thermal degradation and preservation of heat-sensitive volatiles in SPE oils (Gu et al., 2016; Wang et al., 2021). CP and SE samples, located in the lower right quadrant, exhibit some proximity to each other but remain distinct. This proximity suggests that CP and SE share similarities in their volatile profiles, likely due to moderate extraction conditions that preserve certain volatile compounds. SBE, centrally positioned, displays a volatile profile intermediate between those of CP and SPE. Its central location suggests that SBE captures a balanced volatile spectrum, retaining both heat-sensitive and stable compounds.

In summary, PCA results demonstrate that the extraction method significantly influences the oil's volatile profile, with each method imparting a unique volatile compound fingerprint. The clear separation between seeds and oils further suggests that the oil's volatile composition is significantly influenced by both the raw material and the extraction technique.

## 4 Conclusion

FSTOs were extracted using CP, SE, SBE, and SPE methods and were comparatively analyzed for their physicochemical properties, bioactive compounds, and volatile profiles. Oil yields varied notably, with SBE achieving the highest yield (43.25 %), followed by SE, SPE, and CP. The major unsaturated fatty acids—trichosanic acid (9c,11 t,13c-C18,3), linoleic acid (C18:2), and oleic acid (C18:1)—accounted for over 89 % of total fatty acids, with SBE oil showing the highest proportion (92.73 %). CP oil contained the highest levels of phytosterols and flavonoids and exhibited the strongest antioxidant activity, while SBE oil had the highest total phenolic content and oxidative stability. All oils exhibited Newtonian flow behavior; SBE and SPE oils had lower activation energies, suggesting reduced sensitivity to temperature variations. GC-IMS analysis identified 108 VOCs, including 28 alcohols, 19 aldehydes, 19 esters, and 17 ketones. SBE and SPE oils exhibited the most complex and abundant VOC profiles, while CP retained more natural aroma compounds. PCA revealed distinct clustering of the oils based on physicochemical and VOC properties, with SBE oil displaying the most unique characteristics.

Overall, SBE offers the best balance of yield, oxidative stability, unsaturation, and bioactive retention. Its mild conditions, low solvent residue, and energy efficiency make it the most suitable method for environmentally friendly, large-scale production. In addition, SBE is considered safer for the environment, as it employs a legally permitted food processing aid (*n*-butane) that can be fully recovered, thereby preventing harmful solvent emissions. Given their nutritional value, antioxidant capacity, and aroma profiles, FSTOs—especially from SBE and CP—qualify as functional oils for food, nutraceutical, cosmetic, and pharmaceutical applications.

## CRediT authorship contribution statement

**Ling-Biao Gu:** Methodology. **Qiao-Ying Song:** Data curation. **Lin Wang:** Resources. **Xue-Xia Liu:** Supervision, Software. **Wen-Jie Liao:** Investigation. **Rong Gu:** Formal analysis, Data curation. **Hua-Min Liu:** Writing – review & editing, Methodology. **Ya-Ting Zhang:** Methodology, Investigation. **Kun-Peng Zhang:** Project administration, Funding acquisition. **Tian-Xuan Hao:** Resources.

## Declaration of competing interest

The authors declare that they have no known competing financial interests or personal relationships that could have appeared to influence the work reported in this paper.

The author is an Editorial Board Member/Editor-in-Chief/Associate Editor/Guest Editor for this journal and was not involved in the editorial review or the decision to publish this article.

## Data Availability

Data will be made available on request.

## Appendix A Supplementary data

Supplementary material: Fig. S1 3D spectra of VOCs in in seed and oil samples Image 1

## References

[bb0005] Ahangari H., King J.W., Ehsani A., Yousefi M. (2021). Supercritical fluid extraction of seed oils–a short review of current trends. Trends in Food Science & Technology.

[bb0010] Alañón M., Alarcón M., Marchante L., Díaz-Maroto M., Pérez-Coello M. (2017). Extraction of natural flavorings with antioxidant capacity from cooperage by-products by green extraction procedure with subcritical fluids. Industrial Crops and Products.

[bb0015] Amakhmakh M., Hajib A., Belmaghraoui W., Harhar H., Al Abdulmonem W., Goh K.W., Meliani A. (2024). Assessment of the impact of microwave roasting on nutrient content, lipid profile, and oxidative stability of pomegranate seed oil. Food Chemistry: X.

[bb0020] Aruna P., Venkataramanamma D., Singh A.K., Singh R. (2016). Health benefits of punicic acid: A review. Comprehensive Reviews in Food Science and Food Safety.

[bb0025] Delfan-Hosseini S., Nayebzadeh K., Mirmoghtadaie L., Kavosi M., Hosseini S.M. (2017). Effect of extraction process on composition, oxidative stability and rheological properties of purslane seed oil. Food Chemistry.

[bb0030] Dong T., Tian Z., Wang S., Sun J., Chen H., Wang S., Sun B. (2024). Identification of key off-flavor compounds during storage of fried pepper (*Zanthoxylum bungeanum* maxim.) oils by sensory-directed flavor analysis and partial least squares regression (PLSR). Journal of Food Composition and Analysis.

[bb0035] Du M., Gong M., Wu G., Jin J., Wang X., Jin Q. (2024). Conjugated linolenic acid (CLnA) vs conjugated linoleic acid (CLA): A comprehensive review of potential advantages in molecular characteristics, health benefits, and production techniques. Journal of Agricultural and Food Chemistry.

[bb0040] Du M., Jin J., Wu G., Jin Q., Wang X. (2023). Metabolic, structure-activity characteristics of conjugated linolenic acids and their mediated health benefits. Critical Reviews in Food Science and Nutrition.

[bb0045] Farag M.A., Elimam D.M., Afifi S.M. (2021). Outgoing and potential trends of the omega-3 rich linseed oil quality characteristics and rancidity management: A comprehensive review for maximizing its food and nutraceutical applications. Trends in Food Science & Technology.

[bb0050] Gu L.B., Pang H.L., Lu K.K., Liu H.M., Wang X.D., Qin G.Y. (2016). Process optimization and characterization of fragrant oil from red pepper (*Capsicum annuum* L.) seed extracted by subcritical butane extraction. Journal of the Science of Food and Agriculture.

[bb0055] Gu L.-B., Zhang G.-J., Du L., Du J., Qi K., Zhu X.-L., Zhang X.-Y., Jiang Z.-H. (2019). Comparative study on the extraction of *Xanthoceras sorbifolia* Bunge (yellow horn) seed oil using subcritical *n*-butane, supercritical CO_2_, and the Soxhlet method. LWT.

[bb0060] Hou N.-C., Gao H.-H., Qiu Z.-J., Deng Y.-H., Zhang Y.-T., Yang Z.-C., Gu L.-B., Liu H.-M., Zhu X.-L., Qin Z. (2024). Quality and active constituents of safflower seed oil: A comparison of cold pressing, hot pressing, Soxhlet extraction and subcritical fluid extraction. LWT.

[bb0065] Hou Z., Zhu L., Meng R., Wang B. (2020). Hypolipidemic and antioxidant activities of *Trichosanthes kirilowii* maxim seed oil and flavonoids in mice fed with a high-fat diet. Journal of Food Biochemistry.

[bb0070] Islam F., Imran A., Nosheen F., Fatima M., Arshad M.U., Afzaal M., Biswas S. (2023). Functional roles and novel tools for improving-oxidative stability of polyunsaturated fatty acids: A comprehensive review. Food Science & Nutrition.

[bb0075] Ivanova-Petropulos V., Mitrev S., Stafilov T., Markova N., Leitner E., Lankmayr E., Siegmund B. (2015). Characterisation of traditional Macedonian edible oils by their fatty acid composition and their volatile compounds. Food Research International.

[bb0080] Kobori M., Ohnishi-Kameyama M., Akimoto Y., Yukizaki C., Yoshida M. (2008). Α-Eleostearic acid and its dihydroxy derivative are major apoptosis-inducing components of bitter gourd. Journal of Agricultural and Food Chemistry.

[bb0085] Liu H., Wen J., Huang G., Yuan Z., Yang J., Wu J., Yu Y., Hu T., Xu Y. (2022). Assessment of oil extracted from Gardenia fruits by different commercial extraction methods for potential industrial applications. Industrial Crops and Products.

[bb0090] Liu H.-M., Yao Y.-G., Ma Y.-X., Wang X.-D. (2020). Ultrasound-assisted desolventizing of fragrant oil from red pepper seed by subcritical propane extraction. Ultrasonics Sonochemistry.

[bb0095] Liu X.-Y., Ou H., Gregersen H., Zuo J. (2023). Supercritical carbon dioxide extraction of *Cosmos sulphureus* seed oil with ultrasound assistance. Journal of CO_2_ Utilization.

[bb0100] Ma X., Zheng C., Zhou Q., Huang C., Wang W., Huang Y., Liu C. (2024). Comparison evaluation pretreatments on the quality characteristics, oxidative stability, and volatile flavor of walnut oil. Food Chemistry.

[bb0105] Machado M., Rodriguez-Alcalá L.M., Gomes A.M., Pintado M. (2023). Vegetable oils oxidation: Mechanisms, consequences and protective strategies. Food Reviews International.

[bb0110] Miao W.B., Li Y.J., Ma S.Y., Jiang J.H., Liu H.M., Cai X.S., Wang X.D. (2022). Effects of cold-pressing conditions on physicochemical and functional properties of cold-pressed tigernut oil and starch isolated from press-cake. International Journal of Food Science & Technology.

[bb0115] Qin Z., Chang Y.-L., Chen Z.-M., Wang Y.-G., Fan W., Gu L.-B., Qin Z., Liu H.-M., Zhu X.-L., Mei H.-X. (2024). A novel strategy for preparing lignan-rich sesame oil from cold-pressed sesame seed cake by combining enzyme-assisted treatment and subcritical fluid extraction. Industrial Crops and Products.

[bb0120] Shakoor A., Zhang C., Xie J., Yang X. (2022). Maillard reaction chemistry in formation of critical intermediates and flavour compounds and their antioxidant properties. Food Chemistry.

[bb0125] Sumara A., Stachniuk A., Montowska M., Kotecka-Majchrzak K., Grywalska E., Mitura P., Fornal E. (2023). Comprehensive review of seven plant seed oils: Chemical composition, nutritional properties, and biomedical functions. Food Reviews International.

[bb0130] Thilakarathna R., Siow L.F., Tang T.-K., Chan E.-S., Lee Y.-Y. (2023). Physicochemical and antioxidative properties of ultrasound-assisted extraction of mahua (*Madhuca longifolia*) seed oil in comparison with conventional Soxhlet and mechanical extractions. Ultrasonics Sonochemistry.

[bb0135] Wang W.Y., Yan Y.Y., Liu H.M., Qi K., Zhu X.L., Wang X.D., Qin G.Y. (2021). Subcritical low temperature extraction technology and its application in extracting seed oils. Journal of Food Process Engineering.

[bb0140] Wu S.M., Xu T., Akoh C.C. (2014). Effect of roasting on the volatile constituents of *Trichosanthes kirilowii* seeds. Journal of Food and Drug Analysis.

[bb0145] Xi B.-N., Zhang J.-J., Xu X., Li C., Shu Y., Zhang Y., Shi X., Shen Y. (2024). Characterization and metabolism pathway of volatile compounds in walnut oil obtained from various ripening stages via HS-GC-IMS and HS-SPME-GC–MS. Food Chemistry.

[bb0150] Xu L., Liu T., Cao H., Zheng L., Zhu C., Karrar E., Shen X. (2022). Influence of different extraction methods on the chemical composition, antioxidant activity, and overall quality attributes of oils from *Trichosanthes kirilowii* maxim seed. Food Control.

[bb0155] Xu L., Wang S., Tian A., Liu T., Benjakul S., Xiao G., Ying X., Zhang Y., Ma L. (2023). Characteristic volatile compounds, fatty acids and minor bioactive components in oils from green plum seed by HS-GC-IMS. GC-MS and HPLC. *Food Chemistry: X*.

[bb0160] Xu Y., Bi S., Niu X., Chen Y., Liu Y., Zhou Q. (2023). Comparison of aroma active compounds in cold-and hot-pressed walnut oil by comprehensive two-dimensional gas chromatography-olfactory-mass spectrometry and headspace-gas chromatography-ion mobility spectrometry. Food Research International.

[bb0165] Yuan G.-F., Yuan J.-Q., Li D. (2009). Punicic acid from *Trichosanthes kirilowii* seed oil is rapidly metabolized to conjugated linoleic acid in rats. Journal of Medicinal Food.

[bb0170] Zamora R., Hidalgo F.J. (2016). The triple defensive barrier of phenolic compounds against the lipid oxidation-induced damage in food products. Trends in Food Science & Technology.

[bb0175] Zeng W., Liu X., Chao Y., Wu Y., Qiu S., Lin B., Liu R., Tang R., Wu S., Xiao Z. (2024). The effect of extraction methods on the components and quality of *Camellia oleifera* oil: Focusing on the flavor and lipidomics. Food Chemistry.

[bb0180] Zhang H., Yuan Y., Zhu X., Xu R., Shen H., Zhang Q., Ge X. (2022). The effect of different extraction methods on extraction yield, physicochemical properties, and volatile compounds from field muskmelon seed oil. Foods.

[bb0185] Zhang H.-L., Wang Z.-X., Wei S.-S., Liu X.-Q., He J.-B., Zhang W.-N., Du J. (2023). *Trichosanthes kirilowi*i maxim seed kernel oil: The optimization of ultrasound-assisted extraction and evaluation of its potential as a novel biodiesel feedstock. Sustainable Chemistry and Pharmacy.

[bb0190] Zhang L., Chen J., Zhang J., Sagymbek A., Li Q., Gao Y., Du S., Yu X. (2022). Lipid oxidation in fragrant rapeseed oil: Impact of seed roasting on the generation of key volatile compounds. Food Chemistry: X.

[bb0195] Zhang Y., Li X., Lu X., Sun H., Wang F. (2021). Effect of oilseed roasting on the quality, flavor and safety of oil: A comprehensive review. Food Research International.

[bb0200] Zhu B., Li Z., Han T., Yan Y., Li J., Zhang J. (2024). Molecular insights into the dispersibility of asphaltene and crude oil rheological properties under the effect of multi-alkylated aromatic amides. Chemical Engineering Science.

