# Supplementary material for: Comparative evaluation of extraction methods for fragrant semen Trichosanthis oil: Cold pressing, conventional solvent, subcritical n-butane and supercritical CO2
Source: Food Chem X. 2025 Jun 16;29:102670. doi: 10.1016/j.fochx.2025.102670 (PMC12212152; doi:10.1016/j.fochx.2025.102670)
Supplement: Supplementary file 1 — Supplementary material: Fig. S1 3D spectra of VOCs in in seed and oil samples [file mmc1.docx]

**Comparative evaluation of extraction methods for fragrant Semen Trichosanthis oil: Cold pressing, Conventional solvent, Subcritical n-butane and Supercritical CO2**

Ling-Biao Gu^a,c,e^, Qiao-Ying Song^a^, Lin Wang^a^, Xue-Xia Liu^a,*^, Wen-Jie Liao^a^, Rong Gu^a^, Hua-Min Liu^b^, Ya-Ting Zhang^c^, Kun-Peng Zhang^a,*^, Tian-Xuan Hao^d^

^a^ Henan Key Laboratory of Subcritical High-Efficiency Extraction, School of Biological and Food Engineering, Anyang Institute of Technology, 455000 Anyang, China

^b^ College of Food Science and Engineering, Henan University of Technology, 450001 Zhengzhou, China

^c^ Library, Zhengzhou University, 450001 Zhengzhou, China

^d^ College of Safety Science and Engineering, Henan Polytechnic University, 454000 Jiaozuo, China

^e^ Henan Subcritical Biotechnology Co. LTD, 455000 Anyang, China

*** Corresponding authors.

*E-mail addresses:* [liuxuexia1128@126.com](mailto:liuxuexia1128@126.com) (X. -X. Liu); [zhangkunpengag@163.com](mailto:zhangkunpengag@163.com) (K. -P. Zhang).


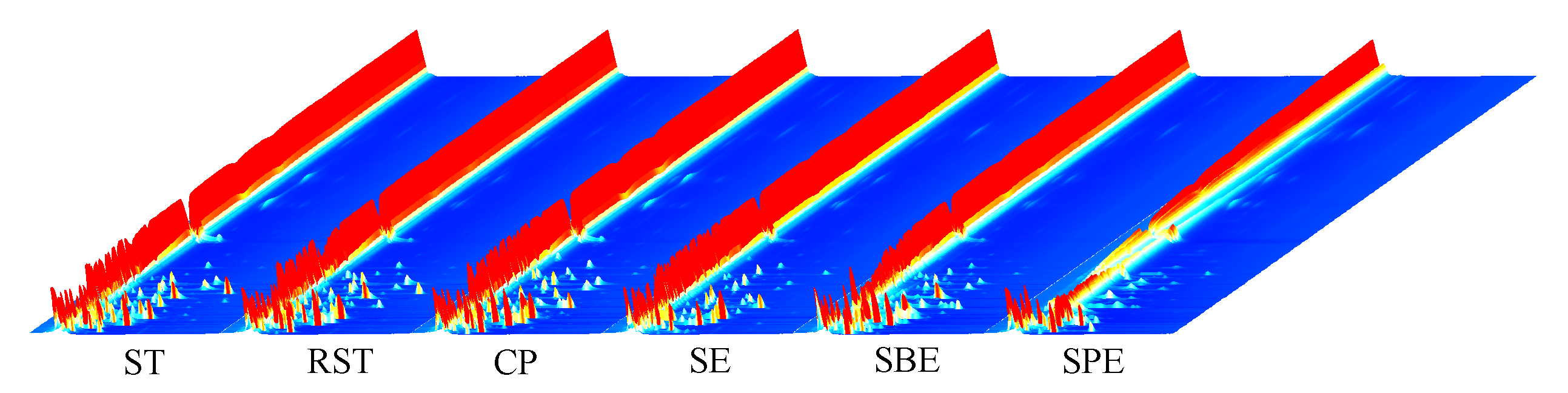


Fig. S1 3D spectra of VOCs in in seed and oil samples.
